# *Hoslundia opposita vahl*; a potential source of bioactive compounds with antioxidant and antibiofilm activity for wound healing

**DOI:** 10.1186/s12906-024-04540-z

**Published:** 2024-06-17

**Authors:** Catherine Namuga, Haruna Muwonge, Kerebba Nasifu, Peter Sekandi, Tahalu Sekulima, John Baptist Kirabira

**Affiliations:** 1 Depatment of Polymer, Textile, and Industrial Engineering, Busitema University, P. O. Box 256, Tororo, Uganda; 2 Department of Physiology, College of Health Sciences, Makerere University, P. O. Box 7062, Kampala, Uganda; 3 Department of Chemistry, College of Natural Sciences, Makerere University, P. O. Box 7062, Kampala, Uganda; 4 Department of Microbiology, College of Veterinary Medicine, Animal Resources and Biosecurity, Makerere University, P. O. Box 7062, Kampala, Uganda; 5 Department of Mechanical Engineering, College of Engineering, Design, Art, and Technology, Makerere University, Kampala, Uganda

**Keywords:** Antibiofilm, Antioxidant, Phytochemicals, GC-MS, Hoslundia opposita vahl, LC-MS

## Abstract

**Background:**

Biofilms and oxidative stress retard wound healing. The resistance of biofilms to antibiotics has led to a search for alternative approaches in biofilm elimination. Antioxidants work synergistically with antibacterial agents against biofilms. Hence recent research has suggested plants as candidates in the development of new alternatives in biofilm treatments and as antioxidants due to the presence of phytocompounds which are responsible for their bioactivities. *Hoslundi*a *opposita Vahl* is one of the plants used by traditional healers to treat wounds and other infections, this makes it a potential candidate for drug discovery hence, in this study, we investigate the antibiofilm and antioxidant activity of methanolic extract of *hoslundia opposita V*ahl from Uganda. We also identify phytochemicals responsible for its bioactivity.

**Method:**

the plant was extracted by maceration using methanol, and the extract was investigated for antioxidant activity using 2,2-diphenyl-1-picrylhydrazyl radical (DPPH) assay. The antibiofilm activity using microtiter plate assay (MTP) assay where the Minimum biofilm inhibitory concentration required to inhibit 50% or 90% of the biofilm (MBIC_50_ and MBIC_90_) and Minimum biofilm eradication concentration required to remove 50% or 90% of the biofilm (MBEC_50_ and MBEC_90_) were measured. It was further analysed for its phytochemical composition using quantitative screening, as well as Gas chromatography-mass spectrometry (GC-MS) and Liquid chromatography mass-spectrometry (LC-MS).

**Results:**

*H. Opposita Vahl* extract showed good antioxidant activity with of 249.6 mg/mL. It inhibited the growth of *P. aeruginosa* and *S. aureus biofilms with* MBIC_50_ of 28.37 mg/mL and 10 mg/mL, respectively. It showed the ability to eradicate *P. aeruginosa* and *S. aureus* biofilms with MBEC_50_ of 23.85 and 39.01 mg/mL respectively. Phytochemical analysis revealed the presence of alkaloids, tannins, flavonoids, and phenols. GC-MS analysis revealed 122 compounds in the extract of which, 23 have evidence of antioxidant or antibiofilm activity in literature. The most abundant compounds were; 1,4- Citric acid, Tetracontane-1,40-diol (43.43.3%, 1, Olean-12-en-28-oic acid, 3-hydroxy-, methyl ester, (3.beta) (15.36%) 9-Octadecenamide (12.50%), Squalene (11.85%) Palmitic Acid 4TMS (11.28%), and alpha Amyrin (11.27%). The LC-MS identified 115 and 57 compounds in multiple reaction mode (MRM) and scan modes respectively.

**Conclusion:**

*H. opposita Vahl* showed antibiofilm and antioxidant activity due to bioactive compounds identified, hence the study justifies its use for wound healing. It can be utilised in further development of new drugs as antibiofilm and antioxidants.

**Supplementary Information:**

The online version contains supplementary material available at 10.1186/s12906-024-04540-z.

## Background

Wounds cause pain and can be life threatening if they take long to heal. Normally, wounds should heal naturally however, infection especially from biofilms can prolong the healing process leading to hospitalisation and financial burden. Biofilms are a group of microorganisms that cling to abiotic and biotic surfaces or a group of microbial aggregates encapsulated within the extracellular polymeric substance (EPS) [1]. Biofilm infections have become a threat in injured and post-surgical patients leading to prolonged wound healing as well as high patient morbidity and mortality. Open wounds are more prone to biofilm infections because they provide a moist and nutrient-rich environment that enables their survival [2]. In the early stages of the wound, microbes are destroyed by the host immune system. However, when these microbes adhere to the surface of the wound and cells, they multiply rapidly through quorum sensing hence forming biofilms [1]. The well-formed biofilm develops resistance (1000 times more) to destruction by the host immune system and antibiotics. Hence cause wound enlargement, difficulty in skin transplants and prolonged healing [1, 2]. This requires specialized management, and not to mention, the huge financial burden in infection management and treatment [1]. National Institute of Health reported that pathogenic biofilms are responsible for about 80% of human tissue infections [3–6], leading to high mortality and health care costs. In the US, 17 million hospital cases and 550,000 deaths due to biofilm infections are reported annually. These account for about 94 billion US dollars per year [6].

Biofilms resist antimicrobial treatments up to 1000 times more than their planktonic counterparts [3, 7]. *Pseudomonas aeruginosa* and *Staphylococcus aureus* are among the common biofilm forming pathogens that cause deadly nosomical infections and are associated with antimicrobial resistance [8]. *S. aureus* biofilms are common in wounds, catheters, and trauma infections while *P. aeruginosa* biofilms are common in wounds [1] and cystic fibrosis lung infections [5]. *P.aeruginosa* has been recorded by the World Health Organisation (WHO) as one of the most deadly bacteria requiring special attention in search and development of new antibiotics [7].

Resistance of biofilms to antibiotics is due to their intrinsic structural properties such as limited permeability of the EPS, production of efflux pumps, and release of inactivating enzymes. They may acquire resistance through gene expression and mutational changes, they obstruct antibiotic permeation, inducing slow growth and adaptive stress reactions as well as persistent cell proliferation [9, 10].

Biofilms have varying physiological and functional properties thus a combination of different therapeutics could be better than a single antibiotic intervention in managing and treating biofilm infections [2]. Hence, recent research has focussed on exploring plants as a potential therapeutic solution to biofilm infections. Plants contain bioactive phytochemicals that work together to prevent antibiotic resistance with minimal side effects [6]. These metabolites such as phenolics, and flavonoids are also a good source of antioxidants, a crucial property in alleviating oxidative stress and wound healing. Reactive oxygen species are produced during the inflammatory phase of wound healing to boost the host immune system against bacterial invasion. However, high levels can lead to oxidative stress through accumulation of free radicals, hence, impair antiprotease substances which safeguard the extracellular matrix and tissue cells. Use of antioxidants prevents or delays oxidative stress through free radical scavenging [11] and plays a role in controlling biofilm formation [12]. Antioxidants work synergistically with antibiotics against resistant microbes and their activity depends on their chemical structure [13].

*Hoslundia opposite Vahl* is a perennial shrub belonging to the Lamiaceae family and the only species in the genus Hoslundia. It is mostly used in subsaharan Africa to treat diseases such as chest pain, stomach pains, wounds, malaria, cystitis, liver diseases, epilepsy, gonorrhoeaa, fever, cough, and sores [14]. In Uganda, it is locally named kamunye (in central region) and is used for cleansing the uterus after birth, treat vaginal laceration, wounds, and other diseases [15]. Research has shown evidence that the plant has potential for wound healing, central nervous depression, possesses antibacterial, antioxidant, hepatoprotective, HIV inhibition, insecticidal [14], anti-anaemic [16] and anticancer properties [17]. The antioxidant activity of this plant from different countries has been investigated forexample, Nigeria [18], Tanzania, Mozambique, Ghana [14], as well as Zimbabwe [19]. However, there is scarce information on the antioxidant activity of the specie from Uganda. Additionally, its antibacterial activity has been investigated [14, 20–24], however, its antibiofilm activity has not yet been explored.

Therefore, in this study, we investigate the antibiofilm and antioxidant activity of methanolic extract of *H. opposita Vahl* from Uganda. We further assess the biochemical composition of the plant using gas chromatography and mass spectroscopy (GC-MS) and liquid chromatography with mass spectroscopy (LC-MS) analysis to identify the possible chemical compounds responsible for its biological activity.

## Materials

Methanol, n-hexane and Dichloromethane and acetonitrile were obtained from Loba Chemicals. Folin-Ciocalteu, gallic acid, DPPH, and rutin were offered by the National chemotherapeutic Research laboratory in Uganda. Dimethyl sulfoxide (DMSO) (99.7%) was obtained from Acros organics. Brain heart infusion (BHI), brain heart infusion supplemented (BHI-S) were from Condalab, while Delbeco’s phosphate buffer was from Lonza. All reagents were analytical grade and all tests were done in triplicate.

## Methods

### Plant preparation

The ariel parts of mature plants of *H. opposita Vahl* were collected from a cattle farmLand in Bbale, Kayunga district in Uganda, in period of November 2021, between 9 am to 12 pm EAT with permission from Ministry of agriculture, animal industry and fisheries Uganda. The plant was identified and authenticated by a botanist Olivia Wanyana at Makerere University Herbarium, department of Botany, where a Voucher specimen (001) was deposited and given accession number 51,245. The plant parts were cleaned, and air dried overnight, then placed in an oven at 27 to 30 °C until they were crispy dry. They were pulverised using an electric grinder into fine powder and kept in airtight containers. The powder was extracted by maceration. Briefly, the powder (100 g) was dissolved in 99% methanol at 1:10 *w/v*. The mixture was kept in an air tight container at room temperature for 7days with frequent agitation [20]. The mixture was filtered through cotton wool and then through Whattman filter paper and the extract concentrated at 40 °C. The obtained extract was stored at 4 °C for further experiments.

### Quantitative phytochemical analysis

#### Estimation of total tannins

The total tannins were determined using the Folin-Ciocalteu method [25] with minor modifications. The sample (0.1 g) was extracted using distilled water (10 mL). 50 µL of the extract was added to a flask containing distilled water (7.5 mL), Folin-Ciocalteu (0.5 mL) and 35% sodium carbonate (1 mL) and the mixture diluted with distilled water to 10 mL, shaken and kept at room temperature for 30 min. Absorbance for the test and standard solutions was measured at 725 nm. The tannin content was expressed in terms of mg/g of tannic acid.

#### Estimation of total polyphenolics

Total phenolic contents in the extracts were also determined using Folin–Ciocalteau method [25]. The sample (0.1 g) was extracted of distilled water (10 mL). Folin–Ciocalteau reagent (0.5 mL) was added to the extract solution (0.1 mL) and total volume adjusted to 8.5 mL with distilled water, kept at room temperature for 10 min, after which of 20% sodium carbonate (1.5 mL) was added. The solutions were incubated in a water bath at 40 ˚C for 20 min and absorbance was measured at 755 nm. The total polyphenolic content was calculated based on the calibration curve and expressed in terms of mg/g of gallic acid.

#### Estimation of total flavonoids

Total flavonoids were estimated using the method of Ordonez et al. [25]. , with modification. The sample (1 g) was extracted in 80% methanol (10 mL). 0.5 mL of 2% AlCl_3_ in ethanol solution was added to 0.1 mL of sample and the absorbance measured at 420 nm after an hour. Total flavonoid content was calculated based on the calibration curve and expressed as Rutin equivalent (RE) milligrams per gram of the extract.

#### Estimation of total alkaloids

The procedure was done as described by Ezenou and coworkers [26] but with slight modification. 200 mL of 10% acetic acid in ethanol was added to the sample (5 g), and left to stand for 4 h. The mixture was filtered, and the extract concentrated in a water bath to one-quarter of the original volume. Concentrated ammonium hydroxide was added until the precipitation was complete. The precipitate was washed with dilute ammonium hydroxide and filtered. The residue is the alkaloid, which was dried and weighed.

#### Determination of antioxidant activity by DPPH scavenging

The procedure was according to Oso et., al [27] but with modification. 0.1 gram of the sample was extracted with 10 mL of methanol overnight and filtered 3.94 mg of 2,2-diphenyl-1-picrylhydrazyl radical (DPPH) were dissolved in 100 mL of methanol. 3 mL of DPPH was added into predetermined volumes of the extract (50, 70, 80, 100 µL) followed by 2 mL of methanol. The same was done, for 1 µg/10µL solution of ascorbic acid (positive control) in methanol. DPPH in methanol was taken as blank. The solutions were rapidly mixed and incubated at 37^0^C for 30 min. The decrease in absorbance was read at 517 nm using at UV/VIS spectrophotometer. The percentage of free radical scavenging (%) activity was calculated by the following formula:

$$free\,radical\,scavenging\,activity={Ac}-{As}/{Ac}\times 100$$

Where Ac = Absorbance of the blank, As = Absorbance of extract or ascorbic acid. The concentration of sample required to scavenge 50% of the DPPH free radical (IC_50_) was calculated by linear regression of the plots where the x-axis represented the various concentrations of the extracts, and the y-axis represented the % inhibition (free radical scavenging activity).

### Inhibition of biofilm formation

The extracts were tested against common biofilm forming pathogens; Standard isolates of *P. aeruginosa* ATCC 27,853 and *S. aureus* ATCC 25,923 from the Microbiology laboratory at the School of Biomedical Sciences, College of Health Sciences, Makerere University were used. The assay was carried out using the microtiter plate assay (MTP) method as described by Kırmusaoğlu (2019) [28] but with some modification. It is a simple high-throughput method used to monitor microbial attachment to an abiotic surfaces [29].

The control cultures were maintained on fresh media after resuscitation using brain heart infusion broth and streaked on Mannitol salt agar for *S. aureus* and king’s medium for *P. aeruginosa*. 100 µL of Mueller Hinton broth supplemented with 1% glucose was dispensed in wells of a sterile 96-well polystyrene microtiter plates. 100 µL of standardized extract (0.5 g/ mL in DMSO) were added in the first wells of the column. Following a 2-fold serial dilution, the extracts were diluted by transferring 100 µL of resultant mixture in the first well to subsequent wells until the last well and 100 µL discarded. One column on each plate was used for a positive control (0.5 mg/mL Ciprofloxacin).

Bacterial suspensions were prepared in brain Heart infusion broth supplemented with 1% glucose. The 24 h old cultures were adjusted to 0.5% McFarland (approximately 1 × 10^8^ CFU/mL) and diluted to 5 × 10^6^ CFU/mL. The suspension (20 µL) was added to each well apart from the negative control wells. One column was a blank, to the other column, the organisms were added with no extract. The microtiter plates were then incubated at 37 °C for 16–24 h to allow biofilm formation. The contents were poured, and plates washed with distilled water to remove unattached cells and media components. The formed biofilms were fixed by incubating at 60 °C for 1 h, and stained with 0.1% Crystal Violet (150 µL). After 15 min, the crystal violet was poured out, plates rinsed with Dulbecco’s phosphate buffered saline and dried. The stained biofilms were resolubilized using of 95% Ethanol (150 µL) and absorbance measured at 620 nm by a microplate reader (Thermoscientific MULTISKAN FC).The percentage reduction (PR) of biofilm production of the isolates was calculated using then following formula [28];

$$\mathbf{PR}=\frac{(\mathbf{O}\mathbf{D}\mathbf{C}-\mathbf{O}\mathbf{D}\mathbf{B}) - (\mathbf{O}\mathbf{D}\mathbf{T}-  \mathbf{O}\mathbf{D}\mathbf{B})}{(\mathbf{O}\mathbf{D}\mathbf{C}-\mathbf{O}\mathbf{D}\mathbf{B})}\times 100$$

**ODC**: Optical density of positive control wells (wells inoculated with test organisms and Mueller Hinton broth but with no extract).

**ODB**: Optical density of negative control wells (blank).

**ODT**: Optical density of wells treated with the extracts. Curve of percent reduction against concentration in mg/mL (3.9, 7.8, 15.6, 31.2, 62.2, 125.5, 250, 500) of the extract that inhibited some of the biofilms was plotted and extrapolated to get the Minimum biofilm inhibitory concentration required to inhibit 50% or 90% of the biofilm (MBIC_50_ and MBIC_90_ respectively).

### Biofilm removal assay

The assay was also done using the MTP assay as described by Kırmusaoğlu (2019) [28] but with modification. 180 µL of sterile Mueller Hinton broth was dispensed in wells of sterile microtiter plates. 20 µL of the standardized bacterial suspensions (5 × 10^6^ CFU/mL) were added to the broth in the wells and incubated at 37 °C for 72 h to allow maximum growth. The contents were discarded, and 200 µL of each dose agent obtained from a 2-fold dilution was dispensed in each well of the plate containing biofilm formed rings. Ciprofloxacin (200 µL) was added into one column as a positive control. The plates were incubated at 37 °C for 24 h and the contents discarded. Plates were washed with distilled water and ethanol. The biofilms in the plate were stained using 0.1% Crystal violet and effect of agents on mature formed biofilms determined as described in the MTP assay above. The minimum concentrations of agents eradicating 50 and 90% of mature biofilm formed (MBEC_50_ and MBEC_90_ respectively) were determined as already described.

### Gas chromatography coupled with Mass Spectrometry (GC-MS)

The extract (2 µl) was dissolved in appropriate HPLC grade diluents of different polarities; n-Hexane, dichloromethane, acetonitrile, and methanol. The solutions were filtered and subjected to GC and MS analysis. The methanol extract was analysed in derivatized and underivatized form. The extract dissolved in acetonitrile was also derivatised. Derivatization was done by sylation using N, O-Bis(trimethylsilyl)trifluoroacetamide (BSTFA) prior to GC-MS analysis so as to covert highly volatile analytes into thermally stable compounds hence enhance separation and detection [30].

GC-MS analysis was performed on Shimadzu Nexis (GC-2030 Triple quadrupole) with capillary column (HP5) fused silica (50 m x 0.25 mm) from Restek. Helium was used as the carrier gas. Analysis conditions were 24.5 min, at 100 °C, 3 min at 235 °C for column temperature and 250 °C for injector temperature. The sample (1 µl) was evaporated in a Splitless injector at 300 °C, run time was 24.5 min. The compounds were identified by gas chromatography coupled with mass spectrometry. Molecular weights and structure of compounds of the test materials were ascertained by the interpretation of mass spectrum of GC-MS using the database of the National Institute Standard and Technology (NIST). The mass spectrum of unknown component was compared with the spectrum of the known components in the NIST library. The obtained chromatograms were redrawn in Microsoft excel.

### Liquid chromatography coupled with Mass spectrometry (LC-MS analysis)

An Agilent 6400 Series Triple Quadrupole LC/MS (LC/MS QQQ Mass Spectrometer) equipped with a Sampler (Model: G7129A), MassHunter Data Acquisition Software for data acquisition was used for analysis in both multiple reaction monitoring (MRM) and full scan MS/MS modes. In the MRM mode, electrospray ionization mode (ESI-MS) mode was carried out in both positive and negative ion modes and a spray voltage of + 3,500 V, an S-lens voltage of 140 V, a vaporizer temperature of 300 °C and post time 2.00 min. Qualifiers, Q1, Q2 and Q3 were set at unit resolution. 200 Dwell time for MRM transitions was set to 20 ms resulting in a cycle time of 1.2 s. Collision energy (CE) was between 10 and 75 eV for M + or [M + H]+, and [M-H]- Q1 → Q3 and Q1 → Q2 and the product ion spectra were recorded using a collision energy (CE) of 150 eV with temperature set at 45.00 °C, normal flow rate of 0.300 mL/min with a binary pump (Model: G1312B) and pressure of 550.00 bar. The maximum flow gradient was 100.000 mL/min² with a total run time of 30 min. The solvent composition was 50% H20 (A) and 50% MeOH (B) while the gradient composition was as follows: 80% A and 20%B for 1 min followed by 50% each of A and B for another 5 min. After 10 min the composition changed to 20%A and 80% B, 5%A and 95% B for another 15 min followed by continuous composition for further 25 min and finally 80% A and 20% B for final 26 min.

In the full scan MS/MS mode (MS1 (Q1) is set at a single m/z and MS2 (Q3) is scanned), the scan time was 500 in the mass range m/z 100–1000, fragment voltage set at 135 v and the polarity was in both positive and negative modes. The column temperature was set at 50.00 °C, normal flow rate of 0.300 mL/min with a binary pump (Model: G1312B) and a pressure of 580.00 bar and maximum flow gradient was 100.000 mL/min², with post time 4.00 min and a total run time of 30 min. The solvent composition was 95% H20 in 20mM ammonium acetate (A) and 5% MeOH in plain methanol (B). The gradient composition was as follows: 70% A and 30%B for 2 min at 0.300 mL/min followed by 35% A and 65%B for another 4 min at 0.400 mL/min. The composition remained to 35%A and 65% B for another 5 min at 0.400 mL/min, after it changed to 5%A and 95% B for 10 min at 0.400 mL/min. It remained at 5%A and 95% B for 20 min at 0.400 mL/min and finally it changed to 95%A and 5% B for 20.5 min at 0.300 mL/min.

## Results

### Quantitative phytochemistry and antioxidant activity

**Fig. 1:**
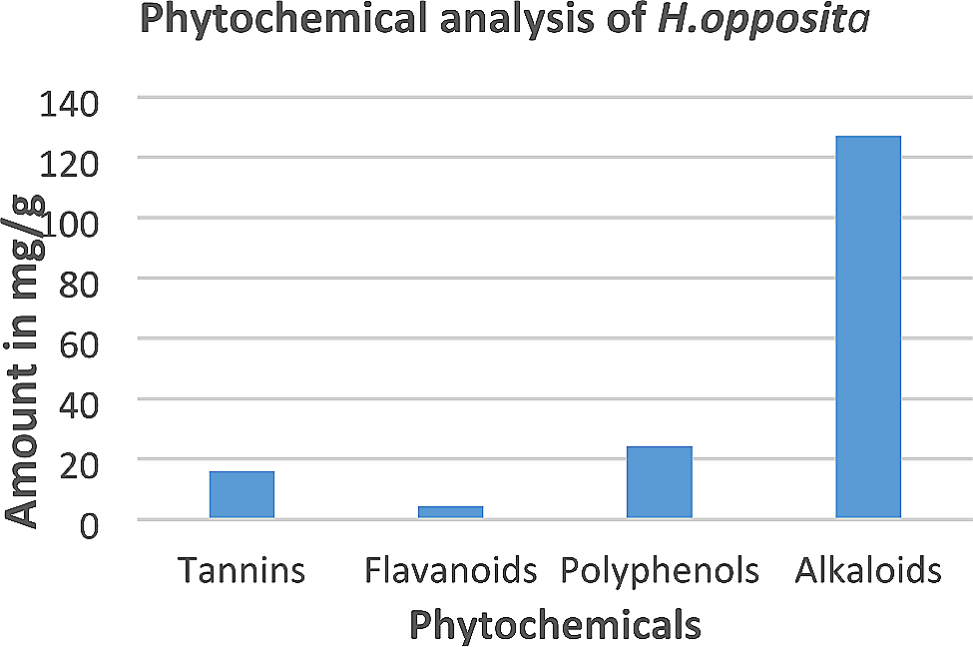
Quantity of total tannins, flavonoids, polyphenols, and alkaloids present in the methanolic extract of H. opposita Vahl

Phytochemicals such as tannins, flavonoids, phenols, and alkaloids are responsible for the bioactivity of plants. The amount of such phytochemicals present in the plant extract are shown in Fig. 1. The quantity of total tannins, total flavonoids, total polyphenols, and total alkaloids was 16 ± 0.696 GAE mg/g, 8.24 ± 0.16 RE mg/g, 24.29 ± 2.24 GAE mg/g, and 127.1 ± 00 GAE mg/g respectively. Alkaloids were the most abundant phytochemicals in this plant.

### Antioxidant activity

The antioxidant activity of the plant was measured against ascorbic acid (a known antioxidant agent) as the standard and the IC_50_ (The concentration of sample required to scavenge 50% of the DPPH free radical) of the plant extract is presented. The positive control Ascorbic acid presented the lowest value of IC_50_ (11.85 µg) and *H. opposita* showed antioxidant activity with IC_50_ of (249.6 mg/mL). According to Abeysuriya et, al [31]. , the IC_50_ between 100 and 500 mg/mL shows good antioxidant activity.

### Antibiofilm activity

**Fig. 2:**
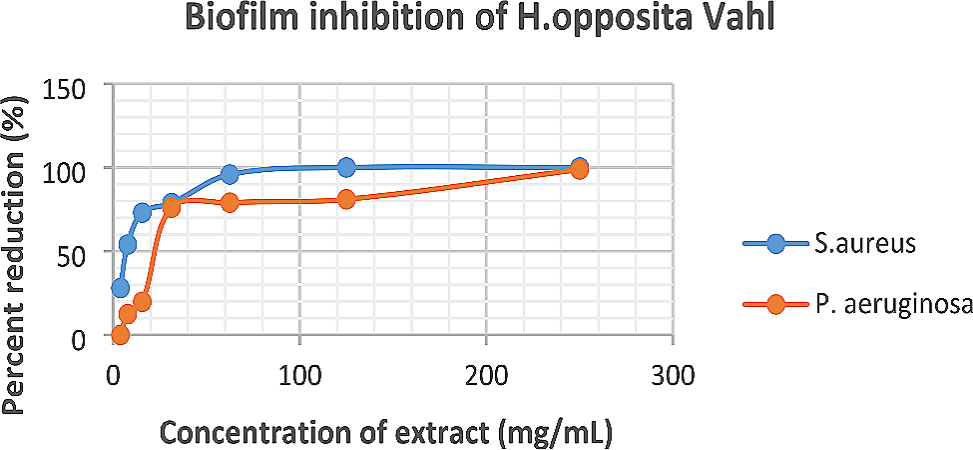
Biofilm inhibition of *H. opposita Vahl* against *S. aureus* and *P. aeruginosa* biofilms

The results of biofilm inhibition by the plant extract are presented in Fig. 2. The equations derived from the curves were *y = 25.388ln(x) − 34.934* and *y = 20.449ln(x) + 2.9089* for *P. aeruginosa* and *S. aureus* respectively. *H. opposita* inhibited *P. aeruginosa* and *S. aureus with* MBIC_50_ of 28.37 mg/mL and 10 mg/mL respectively. The MBIC_90_ was 137.13 mg/mL and 97.039 mg/mL for *P. aeruginosa* and *S. aureus* respectively. *S. aureus* was more susceptible to inhibition by the plant *compared to P. aeruginos*a.

**Fig. 3:**
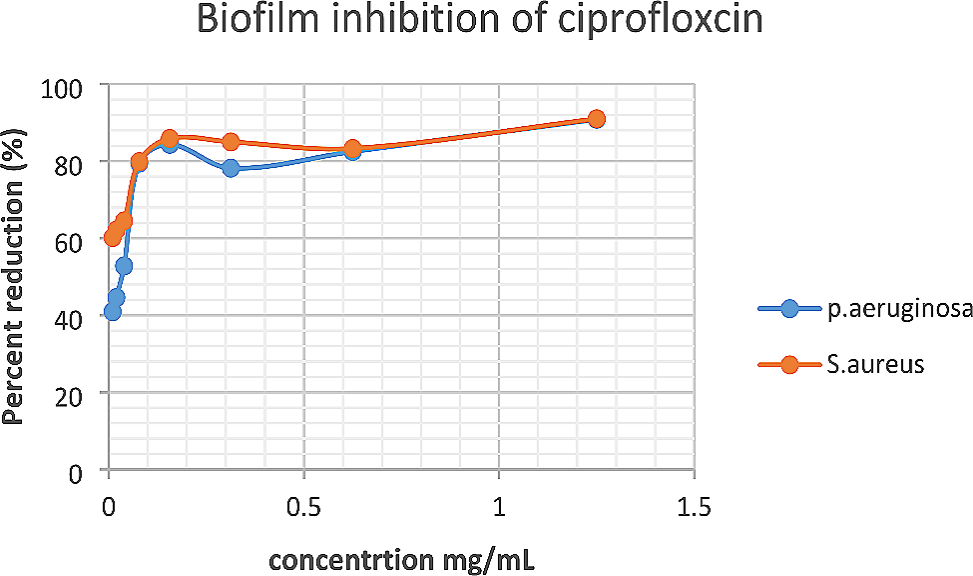
Biofilm removal of *S. aureus* against *P. aeruginosa* biofilms by the methanolic extract of *H. opposita Vahl*

Considering the results of biofilm removal (Fig. 3) the equations obtained from the curve were.

*y = 17.968ln(x) − 6.9916* and *Y = 22.43lnx-32.18* for *P. aeruginosa* and *S. aureus* respectively.

**Fig. 4:**
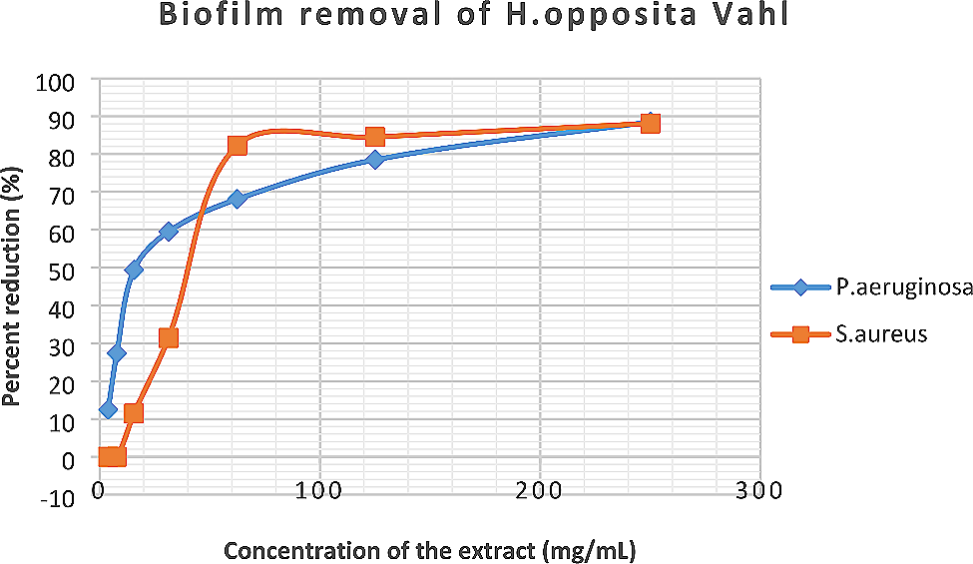
Biofilm inhibition of ciprofloxacin

**Fig. 5:**
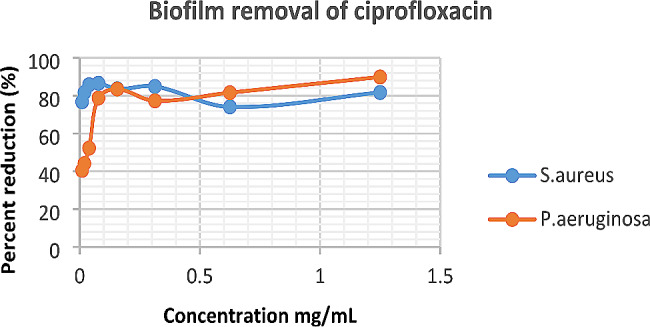
Biofilm removal of *P. aeruginosa* and *S. aureus* by ciprofloxacin

From the results, it is shown that *H. opposita* extract eradicated *P. aeruginosa* and *S. aureus* with MBEC_50_ of 39.01 mg/mL and 23.8.4 mg/mL respectively and MBEC_90_ was 232.1 mg/mL and 220.96 mg/mL respectively. The biofilms formed by *S. aureus* showed more resistance to removal by the plant extract compared to *P. aeruginosa*. The positive control ciprofloxacin inhibited or removed 100% of the biofilm of both *P. aeruginosa*, and *S. aureus* at low concentration of 0.0098 mg/mL (Figs. 4 and 5).

### GC-MS analysis

**Fig. 6:**
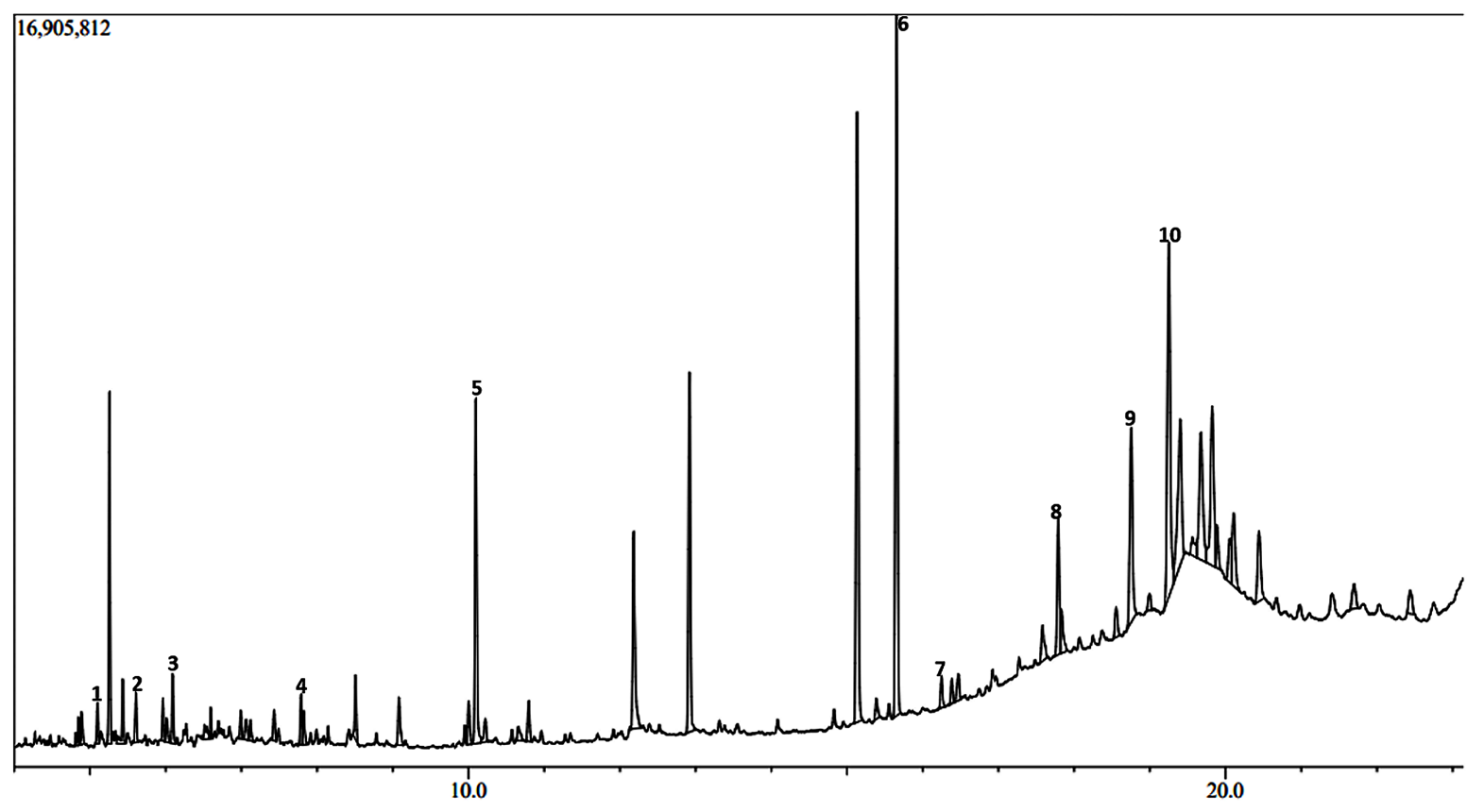
Chromatogram showing some major peaks of *H. opposita Vahl* extract dissolved hexane before GC-MS analysis

**Fig. 7:**
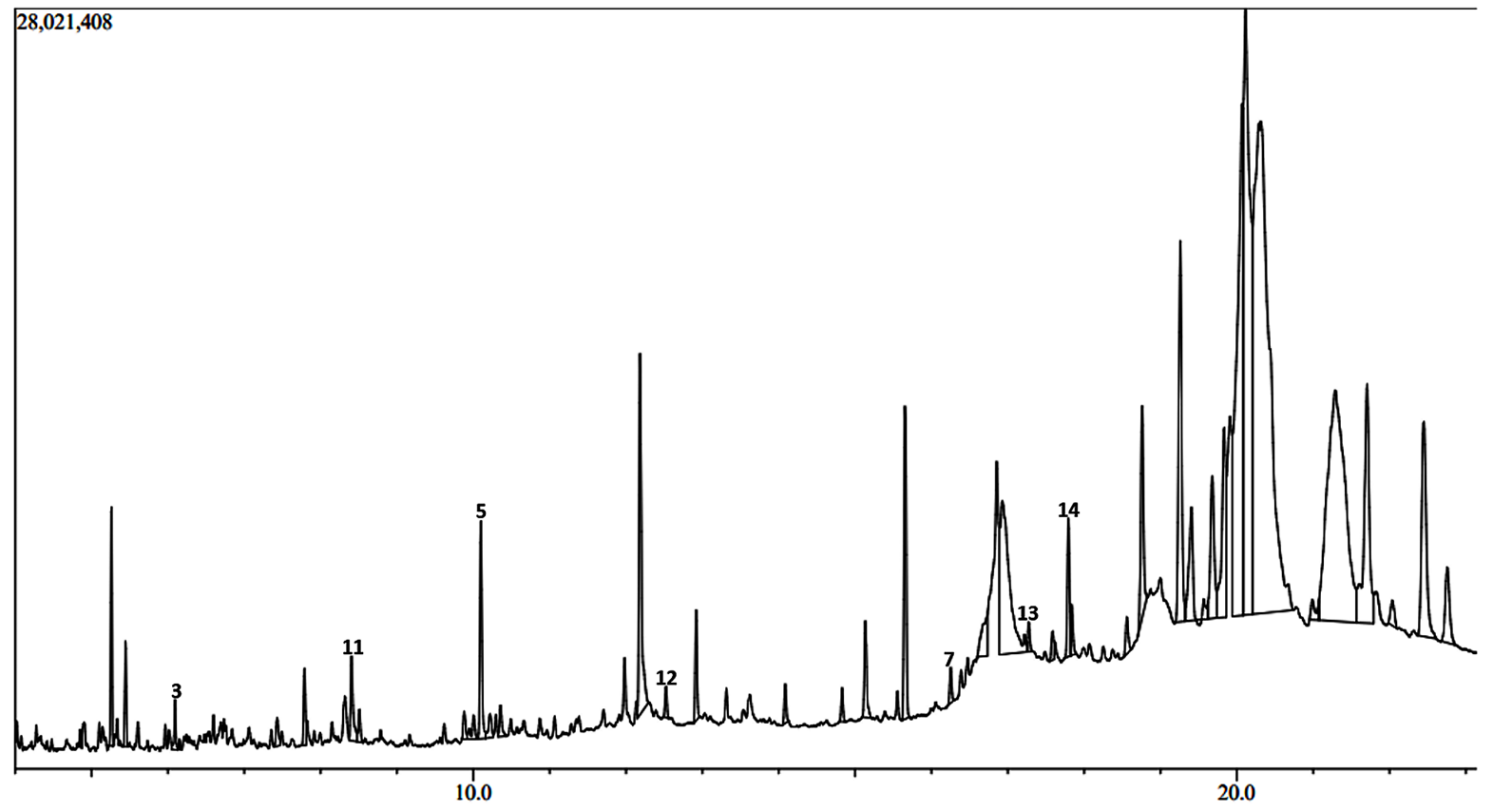
Chromatogram showing some major peaks of *H.opposita Vahl* extract dissolved in Dichloromethane before GC-MS analysis

**Fig. 8:**
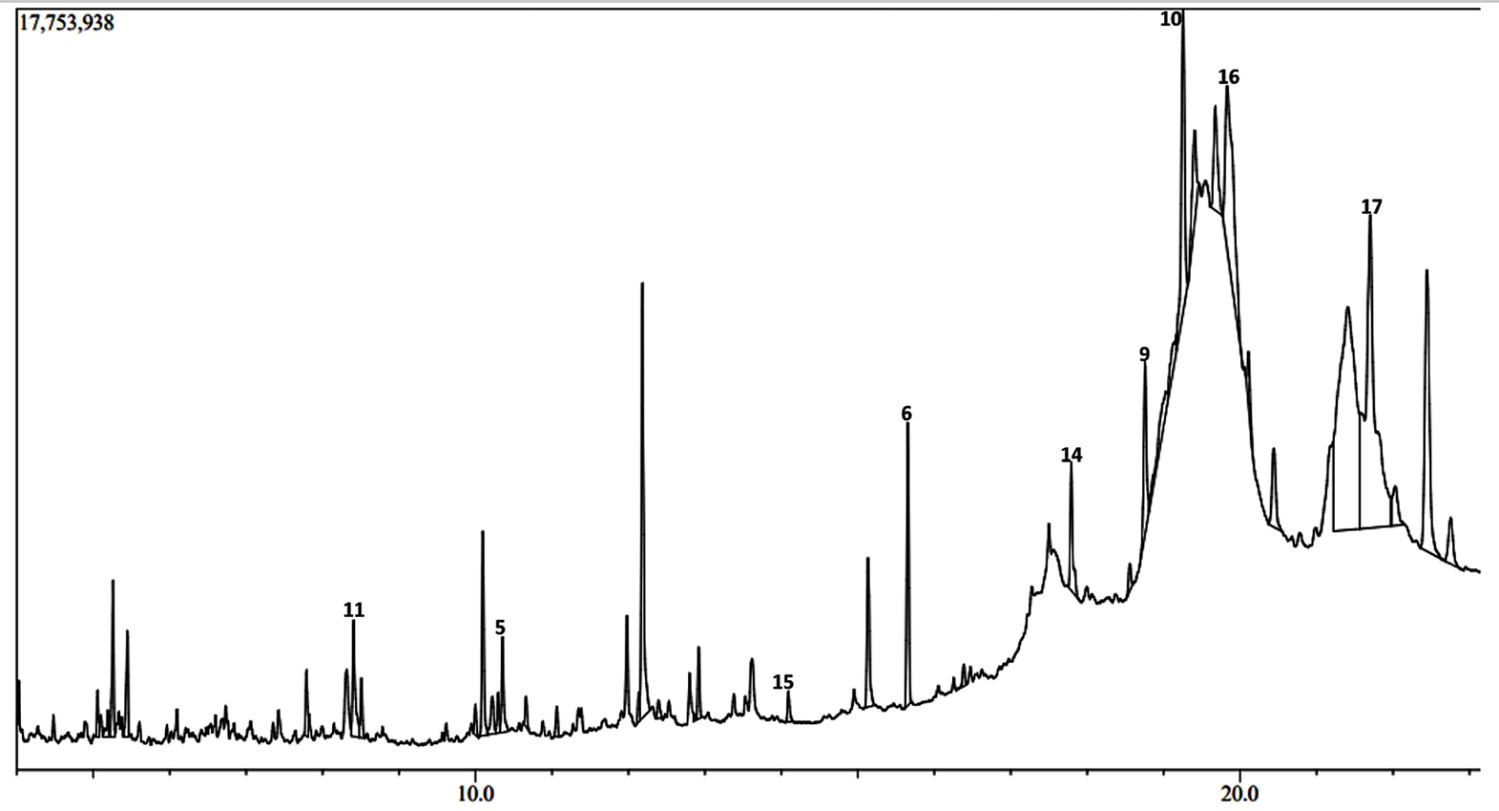
Chromatogram showing some major peaks of *H. opposita Vahl* extract dissolved in methanol before GC-MS analysis

**Fig. 9:**
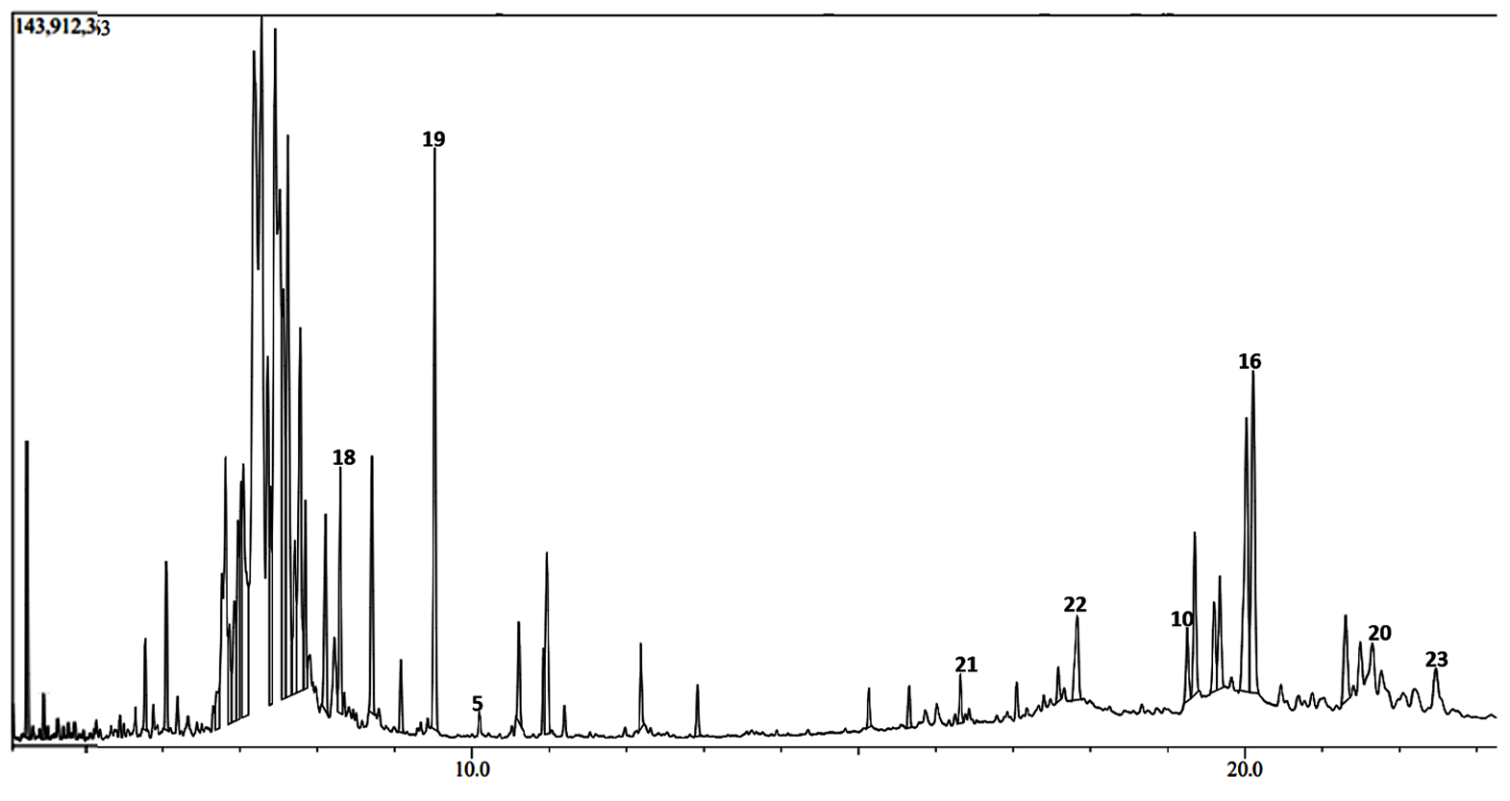
Chromatogram of the *H. opposita Vahl* methanol extract after derivatisation

**Fig. 10:**
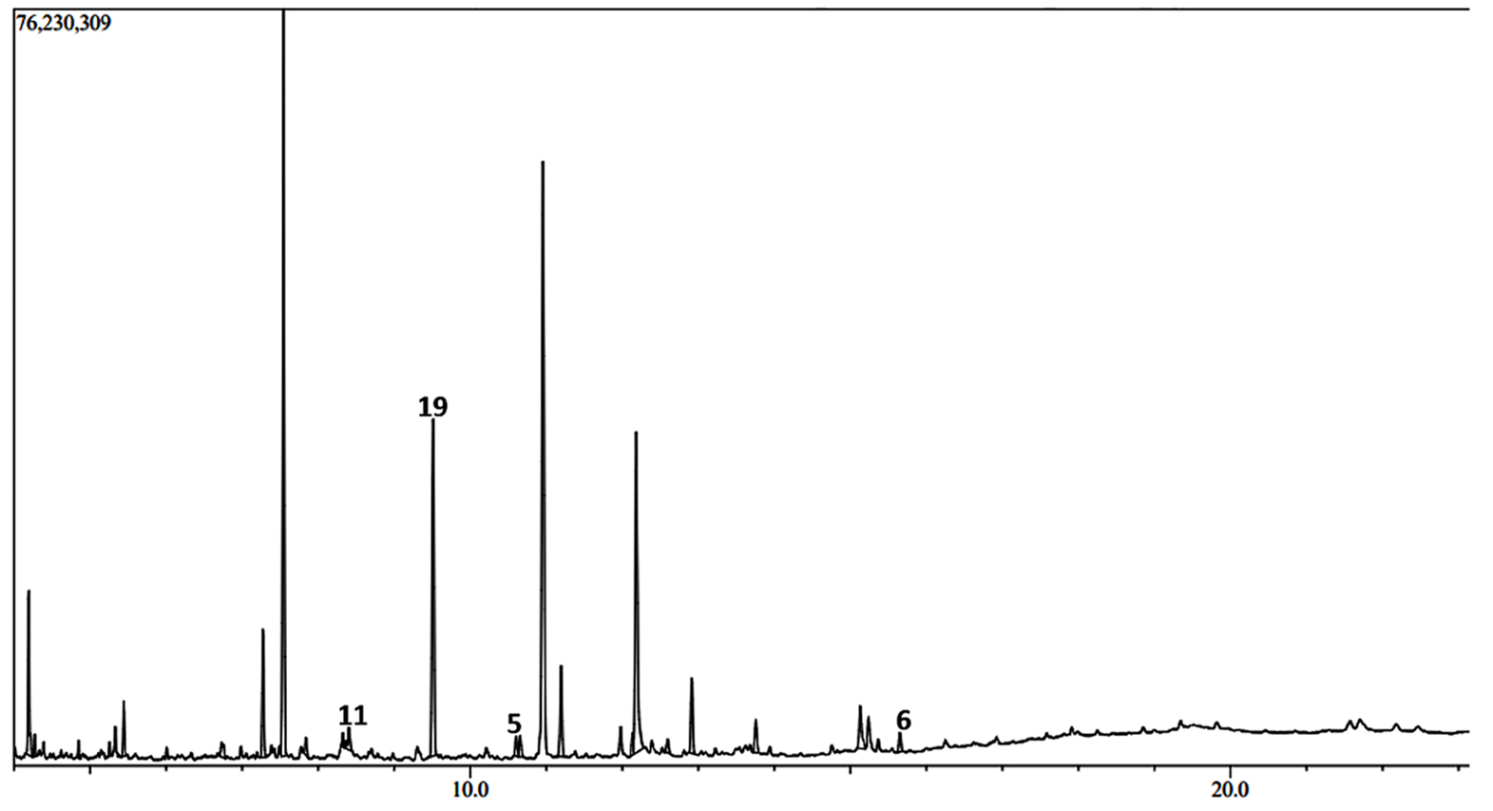
Chromatogram of  *H. opposita Vahl* methanolic extract dissolved in acetonitrile and derivatised before analysis

The GC-MS chromatograms of the *H. opposita* Vahl plant extract are shown in Figs. 6, 7, 8, 9 and 10. The numbered peaks are for identified compounds with evidence of antibiofilm and/or antioxidant activity. The names of these compounds are listed in Table 1, other identified compounds are listed in supplementary Table 1a (additional file 1). Table 1 and supplementary Table 1a (additional file 1) present the compounds identified in each solvent, their molecular formula, molecular weight, retention time, peak area and known biological activities. The chromatograms are shown in Figs. 6, 7, 8, 9 and 10. A total of 122 compounds were identified, the type, concentration and number of the identified compounds depended on the solvent used to dissolve the extract before GC-MS analysis. 47 compounds were identified in hexane, 40, in DCM 47, in methanol, 46 in derivatised methanol and 16 in derivatised acetonitrile. 56 of all the identified compounds have their biological activities reported in literature. as shown in Table 1 and supplementary Table 1a (in additional file). They include; antibacterial, anti-inflammatory, antianalgesic, antiduretic, antimicrobial, hepatoprotective, antiallergic, antiviral, cytotoxic activities, anti-inflammatory, antipruritic effects, spasmolytic activity, anti-angiogenic, antifungal, antioxidant, antibiofilm, anti-HIV, anti-protozoal, antiproliferative, anti-invasive, cholesterol lowering, antispasmodial, antiacne, analgesic, prophylactic, anticarcinogenic, anti-diahorrea, hypocholesterolemic, hepatoprotective, nematicide, insectifuge, antihistaminic, antieczemic, cardioprotective, and antisepsis.

23 of the identified compounds have been reported in literature to have evidence of either antioxidant or/and antibiofilm activity as shown in Table 1. The major compounds (peak area > 5%) are; Citric acid (23.97%), Olean-12-en-28-oic acid, 3-hydroxy-, methyl ester, (3.beta) (15.36%) ), Squalene (11.85%), palmitic Acid 4TMS (11.28%) 1,4-Benzenedicarboxylic acid, bis(2-ethylhexyl)ester (10.29%), Lupeol (7.03%), Phytol (5.74%) gamma-Sitosterol (8.97%).

Derivatisation enabled identification of 39 compounds which were not identified in the underivatized methanol extract. The major compounds were Palmitic acid 4TMS (11.28%), Citric acid (22%), 9-octadecanamide, (Z) (12.5%). Major compounds identified whose biological activity is limited in literature are; 9-octadecenyl ester (Z, Z) (18.77%), Urs-12-en-28-oic acid, 3-hydroxy-, methyl ester (6.34%), Cyclohexane, 1,3,5-triphenyl- (6.26%), and 24-Noroleana-3,12-diene (14.16%). The largest group of compounds were terpenes and terpenoids followed by fatty acids, glycosides and then others.

### LC-MS analysis

**Fig. 11:**
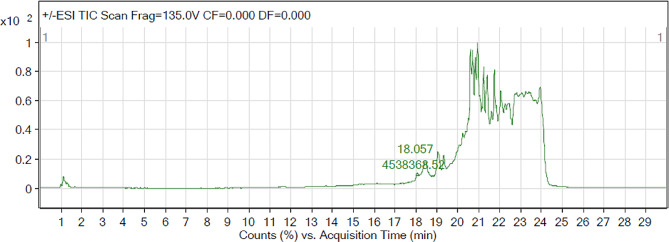
LC-MS Chromatogram for methanol extract of *H. opposita Vahl* extract in SCAN mode

**Fig. 12:**
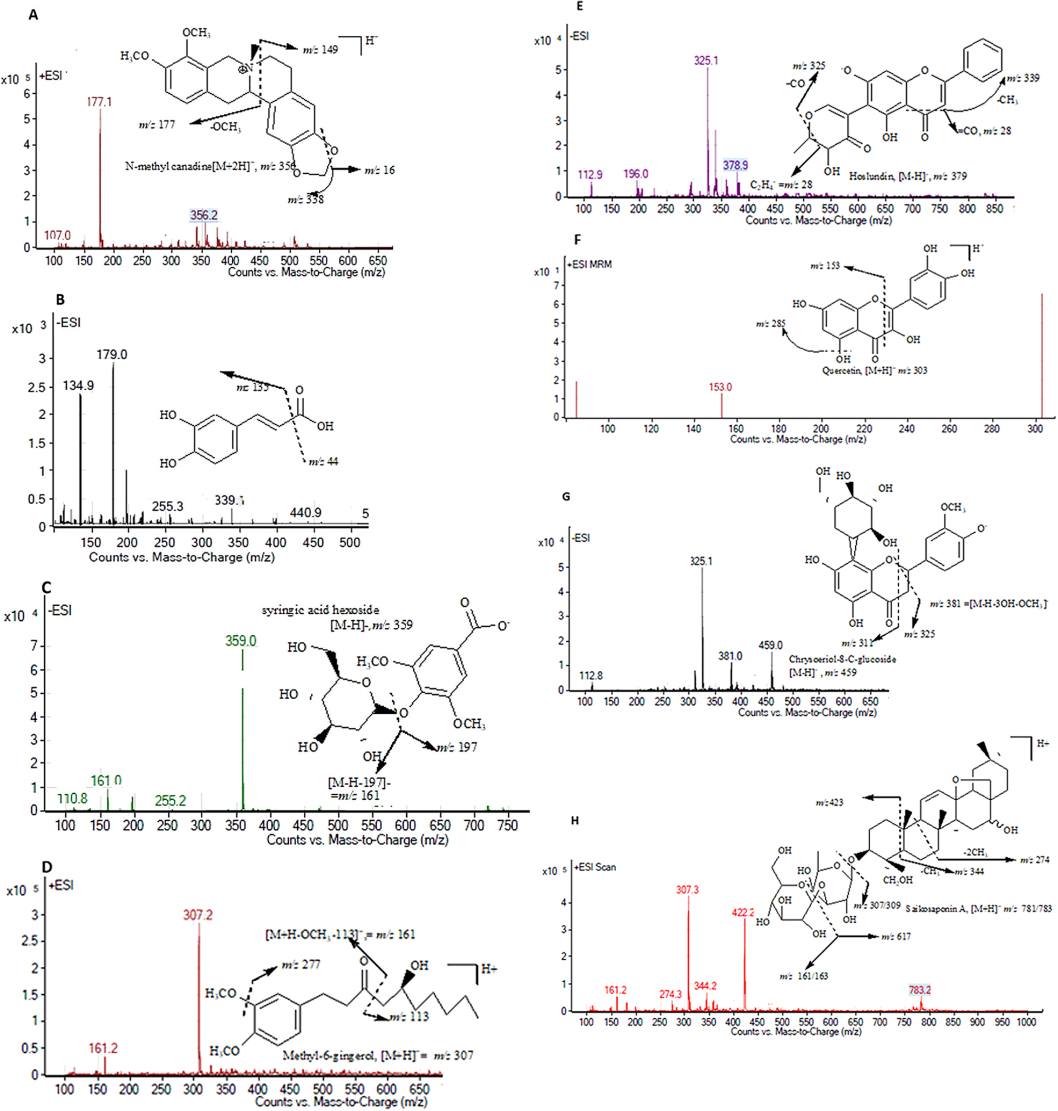
Fragmentation of some compounds; N-methylcanadine (**A**), an alkaloid in positive ion mode, caffeic acid (**B**), a hydroxycinnamic acid in negative ion mode, syringic acid (**C**) hexoside, in negative ion mode, Methyl-6-gingerol (**D**) in negative ion mode, Hoslundin (**E**) an isoflavonoid in negative ion mode, quercetine (**F**) a flavonol in positive ion mode, Chrysoeriol-8-C-glucoside (**G**), a flavone in negative ion mode and saikosaponin A (**H**), a saponin in positive ion mode

LC-MS analysis was done in both MRM and SCAN mode because, the former provides a quantitative approach to identification of phytochemicals and is highly selective and sensitive while the latter is a qualitative and provides a detailed analysis of the mass range. The LC-MS chromatogram in scan mode is shown in Fig. 11 while the fragmentation of some of the identified compounds are shown in Fig. 12. The identified compounds from the LC-MS analysis are shown in supplementary Tables 2 and 3 (additional file 2). A total 115 and 57 phytochemicals were identified from the methanol extract of *H. opposita Vahl* in MRM and SCAN modes respectively.

Alkaloids were the most identified compounds. Other classes of organic compounds identified were; carboxylic acids, hydroxycinnamic acid, flavonols, flavones, flavonones, flavan-3-ols, and coumaric and furanocoumaric, quinolines, aminoacids, acridines, saponins, carboxylic acids.

Among the carboxylic acids, citric acid, isocitric acid, and malic acid were identified. The hydroxycinnamic acids included p-coumaric acid, ferulic acid, P-coumaryl quinic acid, N-trans-feruloyl-tyramine and syringic acid, as well as derivatives such as cinnamoyl quinic acid and caffeic acid hexoside O-pentoside. Some of the flavonoids include flavanones, flavones, flavonols, flavan-3-ols, hosloppin, 3,7-trimethylquercetin, quercetin, eriodictyol, and neohesperidin. The coumarins include simple coumarin and esculetin, while some of the alkaloids were berberine, palmatine, and jatrorrhizine. Several benzophenanthridine type skeleton alkaloids were also identified, including nitidines and chelerythrines. For further details See Additional file 2.

## Discussion

The objective of the study was to determine the biological activity (antioxidant and antibiofilm activity) of the methanol extract of *Hoslundia opposita Vahl* and identify the active compounds that could be responsible for its biological activity using quantitative phytochemical analysis as well as GC-MS and LC-MS analysis. As already mentioned, *Hoslundia opposita* has been used in traditional medicine for wound healing. One of the retarding factors of wound healing is oxidative stress hence use of antioxidants is necessary in management of wounds. Antioxidant activity is very important in accelerating the wound healing process as it safeguards the multiplication of keratinocyte and fibroblasts, as well as prevent the tissue from damage at the wound site which may otherwise be destroyed by excess ROS [27]. From the results, the plant showed good antioxidant activity which justifies its wound healing properties. Our results agree with previous studies that investigated antioxidant activity on the same plant. For example 70% ethanolic extracts of the plant from Nigeria [32], 70%ethanol crude extract of the plant from Zimbabwe [19], and 80% methanol extract from Nigeria [33] all showed good antioxidant activity. The ethanol extract from the Nigerian plant has also been studied for in vivo antioxidant activity and showed positive results [18]. While this plant is useful in treating several illnesses in Uganda, the specie from Uganda has not been reported for antioxidant activity, we report it for the first time and our results further confirm its activity.

Apart from oxidative stress, wound healing is greatly retarded by bacteria infection which occurs when microbes in the wound multiply and produce virulence factors which overpowers the host response and retard the healing process. This becomes worse when bacteria transform from planktonic state to biofilms. The presence of oxidative stress aids bacteria to transform from planktonic state into biofilm as it plays a key role in redox defence mechanisms, biofilm heterogeneity and EPS production [12] which further retards wound healing. The commonest combination of biofilm forming microbes in wound is *P. aeruginosa* and *S. aureus* [34]. *S. aureus* is known to express microbial surface components recognizing adhesive matrix molecules which are capable of binding to human matrix proteins. It also has exceptional ability to stick to plastic or abiotic surfaces compared to other organisms [35]. *P. aeruginosa* poses difficulty with antibiotic treatments due to the presence of efflux pumps and narrow porin channels that make its membrane exceptionally impermeable up to 12 to 100 times lower than other gram-negative bacteria [9]. In mixed biofilms, the two bidirectionally alter their respective antimicrobial susceptibility patterns resulting into severe biofilm infections [36].

Biofilm formation includes attachment, proliferation and maturation which involves gene expression and horizontal gene transfer through quorum sensing [1]. In this study, the plant extract inhibited more than 50% of biofilms formed by the two problematic organisms in microtiter plates. Therefore, it is presumed that the plant compounds could have disrupted the mechanism of attachment and formation of the EPS or interfered with the maturation stage of biofilm formation and quorum sensing, hence inhibition. Inhibiting quorum sensing is a potential solution to bacterial infections because it restricts production of virulence, reducing the risk of antibiotic resistance [9, 37]. The non-permeability of the EPS of biofilms is one of the main reasons for their antibiotic resistance. The ability of *H. opposita vahl* to remove more than 50% of the biofilm is an indication that the phytocompounds in the plant could have interfered with the biochemistry of the EPS and thus were able to eradicate some of the biofilms. The biofilm activity of several plants have been reported elsewhere [5], however this is the first time we report the antibiofilm activity of this plant.

The antioxidant activity and ability of the plant extract to inhibit and eradicate biofilms can be attributed to the presence of flavonoids, alkaloids, phenols, tannins, terpenes and terpenoids as revealed in the phytochemical profiling via quantitative screening, LC-MS (attached file 2) and GC-MS (attached file 1). These phytochemicals are synthesized by plants to act as plant defence mechanisms as well as antimicrobials [38]. Alkaloids and polyphenols are important antioxidants in plants and their antioxidant activities have been explored in many studies.

The bioactive compounds work together and interfere with the biofilm formation as well as scavenge free radicals. Phenolic compounds are hydrogen donors, oxygen scavengers, reducing and metal chelating agents [39] which makes them good antioxidants. Hydroxycinnamic acids have high antioxidant activity, they react with free ROS species to form resonance stabilised phenoxyl radicals. Some of these acids present in the plant such as caffeic, and ferulic acids are strong reducing agents and effective antioxidants [39, 40]. Coumarins identified in LC-MS inhibit formation of ROS and have tissue protective antioxidant activities. Among them is esculetin (6,7-dihydroxycoumarin) which possess antioxidant activity through inhibiting lipoxygenase and cyclooxygenase and lipoxygenase pathways [41]. It is also reported to inhibit biofilm formation *of S. aureus* and *P. aeruginosa* [42]. Elsewhere P-coumaric acid, and ferulic acids prevented biofilm formation against *Escherichia coli* [43].

Flavonoids are well known antioxidants but have also shown evidence as antibiofilm agents. They are reported to inhibit nucleic acid synthesis, and porins on the bacterial cell membrane, altering cell membrane permeability [44]. They prevent synthesis of the extracellular matrix, inhibit virulence factors as well as quorum sensing [45]. Quercetin and its derivatives identified in this study are well known antioxidants and are also reported to inhibit biofilm formation of *P.aeruginosa* by inhibiting quorum sensing and alginate production hence preventing the attachment phase of biofilm formation [45]. Furthermore, Quercetin, neohesperidin, apigenin, and neoeriocitrin acted as autoinducer II inhibitor, causing impaired quorum sensing thus, disrupting formation of *V. harveyi* and *E. coli O157:H7* biofilms [46].

Alkaloids the most abundant compounds in this plant are reported to inhibit biofilm through inhibiting efflux pumps, production of virulence factors as well as quorum sensing [47]. They destroy the cell membrane, affect DNA topoisomerase and respiration [48]. Berberine is one of the alkaloids identified in this study and is known to possess antioxidant, antibiofilm activities among others [49]. Berberine reduced bacterial counts in the in invitro multispecies biofilm (F. nucleatum, P. intermedia and E. faecalis pathogens) [50].

Terpenes and Terpenoids identified by GC-MS are known to inhibit the activity of efflux pumps [51] and lead to uncoupling of oxidative phosphorylation by interfering with cellular respiration within the microbes [52]. Among the terpenes identified is Phytol (5.74%), an acyclic diterpene reported to possess antibiofilm activity against klebsiella pneumoniae and possess anti-virulence factors [53]. Terpenoids identified in the plant with evidence of antibiofilm and/or antioxidant activity are Squalene (11.85%) [54] Olean-12-en-28-oic acid, 3-hydroxy-, methyl ester, (3.beta) (15.36%) [55] and Lupeol (7.03%) [56].

Tannins inhibit synthesis of the bacteria cell wall and chelate ferric iron from the bacterial surrounding, inhibiting their growth. They are also quorum sensing inhibitors and impair gene expression of various virulence factors [57]. Other groups of compounds identified in the plant include fatty acids such as Palmitic Acid 4TMS (11.28%) that has shown evidence of antibiofilm activity against *Vibrio spp* [58]. lignans such as syringaresinol β-D-glucoside, are reported to possess antioxidant activity [59].

*S. aureus* was more susceptible to the plant extract during biofilm inhibition than *P. aeruginosa*. While during biofilm removal, the former was more resistant than the latter. This could be due to the claim that, *S. aureus* strongly adheres to hydrophobic surfaces than to hydrophilic surfaces [60] while *P. aeruginosa* strongly attaches to hydrophilic surfaces than hydrophobic [61]. Therefore, the fact that the assay was performed in a hydrophobic plastic (polystyrene) microtiter plate, these claims could be possible.

During GC-MS analysis, dissolving the extract in different solvents prior to GC-MS analysis helped to identify more compounds, some of which appeared in all solvents but at different concentrations (peak area). More nonpolar compounds were identified in hexane and dichloromethane while more polar compounds were identified in methanol and derivatised samples. For example, squalene is more nonpolar and was 11.85% in hexane solvent as opposed to 2.73% in methanol. Therefore, to extract enough quantity of squalene from this plant, hexane would be the most appropriate solvent. Similarly, alpha amyrin was 11.27% in DCM compared to 0.64% in methanol, hence DCM may be the best solvent to extract it. Results also show the importance of derivatization. Derivatization is a chemical process that modifies compounds to create new products with improved chromatographic properties. In gas chromatography analysis, compounds with functional groups like -COOH, -OH, -NH, and -SH form hydrogen bonds, leading to decreased volatility and thermal stability, as well as potential interactions with column packing. Thus, derivatisation is used for to enhance thermal stability and volatility of compounds, as well as introduce marker molecules for detection purposes, hence improving detectability and separation [62]. In this study derivatisation was done by sylation by substituting the polar compounds with a trimethylsilyl (TMS). Many other compounds and their reported biological activity are shown in supplementary Table 1a additional file 1. The biological activities such as analgensic, anti-inflammatory, antioxidant, antibiofilm, antibacterial are very essential in wound healing. Thus, this justifies why the plant is used by traditional healers for wound healing.

Furthermore, this plant is the only one in the genus hoslundia and this is the first time most of these compounds are identified. There is no study that has done LC-MS analysis on this plant. Previous studies on GC-MS analysis of *H.opposita Vahl* focussed mostly on the identification of compounds in essential oils as opposed to the crude extracts [63, 64]. Of the identified compounds in this study, germacrene D was reported elsewhere to be abundant in essential oils from leaves from Benin, Nigeria, Rwanda, Cameroon, Zimbabwe and Ivory coast while Phytol was abundant in essential oils of the same plant from Nigeria and Ivory coast [63]. In Uganda, the identified compounds in essential oils of this plant that have also been found in our study were; germacrene D, phytol and palmitic acid [64]. Edewor et., al [65] identified compounds from the methanolic extract of *H.opposita Vahl* from Nigeria and found 17 compounds of which Hexadecanoic acid, methyl ester, n-hexadecanoic acid, Octadecanoic acid were also identified in this study. However, the same authors did not find alkaloids and tannins in the quantitative phytochemistry which is opposed to this study. The difference could be due to the difference in geographical location and extraction method.

Among the compounds identified in our study that have been previously isolated from the plant are hosloppin isolated from the methanol extract [66], 5-O-methylhoslundin and hoslundin from methanol extract [67]. Others include oleanolic acid, ursolic acid, sitosterol, and stigmasterol [67].

Generally, LC-MS and GC-MS play an important role in identification of plant phytochemicals. While GC-MS is more suitable for non -polar volatile compounds, LC-MS is suitable for more polar compounds. Using both methods is this study has helped to identify much more compounds that have never been identified before in this plant. Some of the compounds identified, their biological activity is not known in literature however all work together synergistically, or additively to induce antioxidant, antibiofilm and other biological activities which are important for the wound healing ability of the plant and its ability to treat other diseases.

**Table 1:** Suspected compounds identified by GC-MS analysis of methanolic extract of *H. opposita Vahl* that have either antioxidant and or antibiofilm activity. (To be placed in the result section for GC-MS analysis)

Peak No	Suspected compound	Class of compound	Molecular Formula	Molecular weight	Retention time	Hexane (peak area)	DCM (peak area)	Methanol (peak area)	Der. Methanol (peak area)	Der. Acetonitrile (peak area)	Biological activity
1	Caryophyllene	Sesquiterpene	C_15_H_24_	204.3511	5.101	0.48					Antibiofilm [68]Antibacterial, anti-inflammatory, antioxidant activities, anticancer [69], antifungal, analgesic and anti-inflammatory activities [70]
2	1-Nonadecene	Alkene	C_19_H_38_	266.5050	5.964	0.50					Antioxidant [71] Antibacterial and antifungal [72]
3	Caryophyllene oxide	Sesquiterpenoid	C_15_H_24_O	220.3505	6.092	0.94	0.24				antianalgensic and anti-inflammatory, antibacterial [60].
4	Neophytadiene	Diterpene	C_20_H_38_	278.5157	7.789	0.77					anti-inflammatory, antioxidant and cardioprotective [61].
5	Phytol	Diterpenoid	C_20_H_40_O	296.5310	10.075	5.74	1.27	2.02	3.90	0.72	Antibiofilm activity [53], Anti-inflammatory, anticancer, diuretic [73]. and antioxidant [74]
6	Squalene	Triterpene	C_30_H_50_	410.72	15.654	11.85		2.73	0.26	0.71	antibacterial anti-inflammatory, antidiabetic [75], antioxidant, detoxifying and anticancer activity [54].
7	Tetrapentacontane	Alkane	C_54_H_110_	759.4512	16.252	0.57	0.21				Antioxidant and antimicrobial activity [76]
8	Vitamin E	Tocopherol	C_29_H_50_O_2_	430.7061	17.791	2.66					Anti-oxidant activity [77]
9	Stigmasterol	Phytosterol	C_29_H_48_O	412.6908	18.756	4.44	1.64	2.41			prevention of certain cancers, including ovarian, prostate, breast, and colon cancers [73]. antioxidant, antidiabetic and antimicrobial [78]
10	gamma.-Sitosterol	Steroid	C_29_H_50_O	414.7067	19.253	8.97	3.25	4.79	0.60		anti-oxidant, anti-bacterial and prophylatic activities [79] and antidiabetic [80].
11	7,9-Di-tert-butyl-1-oxaspiro (4,5)deca-6,9-diene-2,8 dione	Flavanoid	C_17_H_24_O_3_	276.3707	8.405		0.62	1.58		0.99	Antioxidant activity [81, 82]. Antibacterial [81]
12	Dotriacontane	Alkane	C_32_H_66_	450.8664	12.524		0.17				Antimicrobial, antioxidant, antispasmodial [83].
13	gamma.-Tocopherol	Tocopherol	C_28_H_48_O_2_	416.68	17.275		0.17				Antioxidant and antiinflammatory [84]
14	dl-.alpha.-Tocopherol	Tocopherol	C_29_H_50_O_2_	430.7061	17.795		0.93	1.62			Antioxidant and anti-inflammatory [84]
15	4 H-1-Benzopyran-4-one, 5-hydroxy-7-methoxy-2-phenyl-	Flavone	C_16_H_12_O_4_	268.2641	14.094			0.33			Antioxidant [85]
16	Lupeol	Triterpenoid	C_30_H_50_O	426.7	(20.06)			7.03	3.42		Antioxidant, anti-inflammatory, anti-microbial, anti-protozoal, antiproliferative anti-invasive, anti-angiogenic and cholesterol lowering agent [56].
17	Olean-12-en-28-oic acid, 3-hydroxy-, methyl ester, (3. Beta)	Triterpenoid	C_24_H_61_O_9_PSi_6_	693.2225	21.701			15.36			Antioxidant [86] antimicrobial, hepatoprotective, anti-inflammatory, antiallergic, antiviral and cytotoxic activities. anti-inflammatory, antipruritic effects, spasmolytic activity, anti-angiogenic activities, antiallergic, antiviral [55].
18	Gallic acid, 4TMS	Phenolic acid	C_19_H_38_O_5_Si_4_	458.8440	8.706				1.80		Antibiofilm activity [87] Anti-inflammatory activity [88]. Antioxidant [89],
19	Palmitic Acid TMS	Fatty acid	C_19_H_40_O_2_Si	328.6052	9.519				3.90	11.28	Antibiofilm [58], Inhibition of HIV [30].
20	Betulin	Triterpene	C_30_H_50_O_2_	442.7168	21.648				0.88		Antibiofilm [90] anti-HIV, anti-inflammatory [91] and anticancer [92]
21	Epigallocatechin 6TMS	Flavan-3-ol	C_33_H_62_O_7_Si_6_	739.3542	16.319				0.54		Antibiofilm activity [93] antibacterial and Antifungals [94], Antioxidant Activity [95].
22	alpha-Tocopherolquinone	Quinone	C_29_H_50_O_3_	446.7	17.585				0.97		Antioxidant [96],
23	Ursolic acid 2TMS	triterpene	C_36_H_64_O_3_Si_2_	601.0626	22.469				0.42		Antibiofilm [97] anti-cancer, anti-inflammatory, hepatoprotective, antiallergic and anti-HIV properties [98].

## Conclusion

Antioxidant and antibiofilm activity are important properties in wound healing. The results showed that the methanol extract of *H. opposita Vahl* has both biological properties which justify its use in traditional medicine as a wound healing plant.

The plant contains variety of compounds identified which are biologically active and could be responsible for its antioxidant, antibiofilm and other biological activities. Therefore, the plant can be further be explored for use in development of antibiotics for wound healing and other infections.

### Electronic supplementary material

Below is the link to the electronic supplementary material.

Supplementary Material 1

Supplementary Material 2

## Data Availability

All data is available from corresponding author on reasonable request.

## Abbreviations

ROS: Reactive oxygen species
MBEC: Minimum biofilm eradication concentration
MBIC: Minimum biofilm inhibition concentration
EPS: Extracellular polymeric substance
WHO: World Health Organisation
MRM: Multiple reaction monitoring
NIST: National Institute Standard and Technology
MTP: Microtiter Plate
GC-MS: Gas chromatography-Mass spectroscopy
LC-MS: Liquid chromatography-Mass spectroscopy
