# Supplementary material for: Hoslundia opposita vahl; a potential source of bioactive compounds with antioxidant and antibiofilm activity for wound healing
Source: BMC Complement Med Ther. 2024 Jun 17;24:236. doi: 10.1186/s12906-024-04540-z (PMC11181642; doi:10.1186/s12906-024-04540-z)
Supplement: Supplementary file 1 — Supplementary Material 1 [file 12906_2024_4540_MOESM1_ESM.docx]

**ADDITIONAL FILE ONE**

**Table 1a; Suspected compounds identified by GC-MS analysis of methanol extract of Hoslundia opposita Vahl using different diluents.**

| **No** | **suspected Compound** | **Group of compounds** | **Molecular Formula** | **molecular weight** | **RT** | **Hexane** | **DCM** | **MEOH** | **MEOH derivatized** | **Acetonitrile derivatized** | **Biological activity** |
| --- | --- | --- | --- | --- | --- | --- | --- | --- | --- | --- | --- |
| 1 | Octadecane, 1-chloro- | Chloroalkane | C_18_H_37_Cl | 288.939 | 4.851 | 0.38 |  |  |  |  | antisepsis [1] |
| 2 | Cyclohexane, 1-ethenyl-1-methyl-2,4-bis(1-methylethenyl) | sesquiterpene | C_15_H_24_ | 204.3511 | 4.891 | 0.45 |  |  |  |  | Anti-tumor, antibacterial, anti-inflammatory, analgesic, fungicidal.[2] |
| 4 | Precocene I | *Chromene* | C_12_H_14_O_2_ | 190.2384 | 5.262 | 3.96 | 0.88 | 1.32 |  |  | inhibits Juvenile Hormone biosynthesis.[3] |
| 5 | Germacrene D | *Sesquiterpene* | C_15_H_24_ | 204.3511 | 5.432 | 0.97 |  |  |  |  |  |
| 6 | 1-Isopropyl-4,7-dimethyl-1,2,3,5,6,8a-hexahydronaphthalen | Sesquiterpenoids | C_15_H_24_ | 204.3511 |  | 0.67  (5.607) |  |  |  |  |  |
| 7 | Heptadecane, 2,6,10,15-tetramethyl- | Alkanes | C_21_H_44_ | 296.5741 | 6.013 | 0.43 |  |  |  |  | Ant-HIV and anticancer [4] |
| 8 | Neointermedeol | *Sesquiterpenoid* | C_15_H_26_O | 222.3663 | 6.596 | 0.40 |  |  |  |  | Antimicrobial [5] |
| 9 | Cyclohexane, 1,3,5-triphenyl- | Aromatic hydrocarbon | C_9_H_18_ | 126.2392 | 6.992 | 0.37 |  |  |  |  |  |
| 10 | Tetrapentacontane, 1,54-dibromo- | Alkanes | [C_54_H_108_Br_2_](https://pubchem.ncbi.nlm.nih.gov/#query=C54H108Br2) | 917.2 | 7.063 | 0.40 | 4.17 |  |  |  |  |
| 11 | 1,2-Diphenylcyclopropane | cyclopropane | C_15_H_14_ | 194.2717 | 7.118 | 0.33 |  |  |  |  |  |
| 12 | 2-Pentadecanone, 6,10,14-trimethyl- | *Sesquiterpene* | C_18_H_36_O | 268.4778 | 7.825 | 0.49 |  |  |  |  |  |
| 13 | Hexadecanoic acid, methyl ester | *Fatty* a*cid* | C_17_H_34_O_2_ | 270.4507 | 8.506 | 0.91 | 0.50 | 0.58 |  |  | inhibitor of phospholipase and an anti-inflammatory compound [1] |
| 14 | Hexadecanoic acid, ethyl ester | Fatty acid | C_18_H_36_O_2_ | 284.4772 | 9.082 | 0.91 |  |  | 0.41 |  |  |
| 15 | 9,12,15-Octadecatrienoic acid, 2,3-dihydroxypropyl ester, (Z,Z,Z) | Fatty acid | C_19_H_32_O_2_ | 292.4562 | 10.005 | 0.70 |  |  |  |  |  |
| 16 | Methyl stearate | *fatty acid* | C_19_H_38_O_2_ | 298.5038 | 10**.**223 | 0.48 |  |  |  |  |  |
| 17 | Hexadecanamide | *fatty amide* | C_16_H_33_NO | 255.4393 | 10.655 | 0.36 |  | 0.25 |  | 0.71 |  |
| 18 | Octadecanoic acid, ethyl ester | *fatty acid* | C_20_H_40_O_2_ | 312.5304 | 10.794 | 0.67 |  |  |  |  | Anti-inflammatory, hypocholesterolemic, cancer preventive, antieczemic ,hepatoprotective, nematicide, antihistaminic, insectifuge, antiacne , 5-Alpha reductase inhibitor, antiandrogenic, antiarthritic, anticoronary, and antipsychotic [6] |
| 19 | Cyclohexane, 1,3,5-triphenyl- | Aromatic hydrocarbon | C_9_H_18_ | 126.2392 | 12.919 | 6.26 | 0.62 | 0.73 | 1.05 | 2.65 |  |
| 20 | 9-Octadecenamide, (Z)- | *Fatty acid* | C_18_H_35_NO | 281.4766 | 12.183 | 3.72 | 2.42 | 4.81 | 0.49 | 12.50 | antimicrobial and anti-inflammatory.  Anticancer.[7] |
| 21 | 1,4-Benzenedicarboxylic acid, bis(2-ethylhexyl)ester | Phthalate esters | C_24_H_38_O_4_ | 390.5561 | 15.135 | 10.29 | 0.61 | 1.70 | 0.24 | 1.99 | Anticancer biomarker. [8] |
| 22 | 13-Docosenamide, (Z)- | Amide | C_22_H_43_NO | 337.5829 | 15.390 | 0.39 |  |  |  |  |  |
| 23 | Tetrapentacontane | Alkane | C_54_H_110_ | 759.4512 | 16.252 | 0.57 | 0.21 |  |  |  |  |
| 24 | 1,6,10,14-Hexadecatetraen-3-ol, 3,7,11, ,15-tetramethyl-,(E, E)- | *Sesquiterpene* | C_20_H_34_O | 290.4834 | 16.386 | 0.40 |  |  |  |  | Antitumor, analgesic, antibacterial. [9] |
| 25 | Oxirane ,2,2-dimethyl-3- (3,7,12,16,20-pentamethyl3,7,11,15,19,-henicosapentaenyl | Unsaturated fatty acid | C_20_H_34_O | 290.4834 | 16.471 | 0.58 |  |  |  |  |  |
| 26 | Campesterol | *Phytosterol* | C_28_H_48_O | 400.6801 | 18.561 | 0.69 | 0.29 | 0.34 |  |  | Antiangiogenic, anticarcinogenic effects, cholesterol lowering [10] |
| 27 | 24-Noroleana-3,12-diene | Triterpene | C_29_H_46_ | 394.6755 | 19.40 | 5.00 | 1.29 | 2.05 |  |  |  |
| 28 | 1H-3a,7-Methanoazulen-6-ol, octahydro-3,6,8,8-tetramethyl-, acetate | Sesiqueterpene | C_17_H_28_O_2_ | 264.4030 | 19.56 | 1.54 | 0.19 |  |  |  |  |
| 29 | 24-Noroleana-3,12-diene | Triterpene | C_29_H_46_ | 394.6755 | 19.675 | 5.00 | 2.39 | 2.10 |  |  |  |
| 30 | 24-Noroleana-3,12-diene | Triterpene | C_29_H_46_ | 394.6755 | 19.829 | 5.02 | 2.39 |  |  |  |  |
| 31 | Lanosterol | *Triterpenoid* | C_30_H_50_O | 426.7174 | 20.050 | 1.19 |  |  |  |  | cataract prevention and treatment  [11] |
| 32 | alpha.-Amyrin | *Triterpenoid* | C_30_H_50_O | 426.7 | 20.111 | 1.95 | 11.27 | 0.64 |  |  | Analgesic and anti-inflammatory  [12] |
| 33 | Cholest-4-en-3-one | Cholestanoid | C_27_H_44_O | 384.6377 | 20.441 | 2.05 |  | 1.50 |  |  |  |
| 34 | Urs-12-en-28-oic acid, 3-hydroxy-, methyl ester | *Triterpene* | [C_30_H_48_O_3_](https://pubchem.ncbi.nlm.nih.gov/#query=C30H48O3) | 456.7 | 21.698 | 0.74 |  |  |  |  |  |
| 35 | Urs-12-en-28-oic acid, 3-hydroxy-, methyl ester | *Triterpene* | [C_30_H_48_O_3_](https://pubchem.ncbi.nlm.nih.gov/#query=C30H48O3) | 456.7 | 22.445 | 0.70 | 2.88 | 6.34 |  |  |  |
| 36 | Tetracontane-1,40-diol | Alkane | C_40_H8_2_O_2_ | 595.1 | 22.495 | 2.51 | 22.33  20.311 |  |  |  | Anti-inflammatory activity. [13] |
| 37 | Phenol, 2,5-bis(1,1-dimethylethyl)- | Phenol | C_14_H_22_O | 206.3239 | 5.446 |  | 0.48 | 1.00 |  |  | antibacterial and anti-inflammatory  [14] |
| 38 | 9-Tricosene, (Z)- | Alkene | C_23_H_46_ | 322.6 | 7.433 |  | 0.21 |  |  |  |  |
| 39 | Clonitazene | Opoid | C_20_H_23_CIN_42_ | 386.9 | 7.790 |  | 0.42 | 0.64 |  |  | Analgesia activity [15] |
| 40 | Behenic alcohol | Fatty alcohol | C_22_H_46_O | 326.60 | 9.883 |  | 0.18 |  |  |  | Antiviral, antibacterial, antiacne, anti-inflammatory. [16] |
| 42 | 9 12 15-octadecatrienoic acid 2 3-dihydroxypropyl ester | Fatty acid ester | C_21_H_40_O_4_ | 356.5399 | 10.007 |  | 0.22 |  |  |  | Antibacterial activity. [17] |
| 43 | Ethanol, 2,2'-(dodecylimino)bis- | ehtanolamine | C_16_H_35_NO_2_ | 273.4546 | 10.355 |  | 0.22 | 1.09 |  |  | Anti-inflammatory and antibacterial [18] |
| 44 | Cyclohexane, 1,3,5-triphenyl- | Aromatic hydrocarbon | C_9_H_18_ | 126.2392 | 12.75 |  | 0.62 | 0.64 |  |  |  |
| 45 | 2,6,10,15,19,23-Pentamethyl-2,6,18,22  (2,6,10,15,19,23-Pentamethyl-2,6,18,22-tetracosatetraen-10,15-diol ) | Polyene | C_30_H_54_0_2_ | 446.7 | 15.656 |  | 1.83 |  |  |  |  |
| 46 | Eicosyl nonyl ether | Nonyl ether | C_29_H_60_O | 424.7861 | 16.254 |  | 0.67 |  |  |  |  |
| 47 | Tetracontane-1,40-diol | Aliphatic diol | [C_40_H_82_O_2_](https://pubchem.ncbi.nlm.nih.gov/#query=C40H82O2) | 595.1 | 20.065 |  | 9.34 |  |  |  | Anti-inflammatory activity. [13] |
| 48 | Tetracontane-1,40-diol | Aliphatic diol | [C_40_H_82_O_2_](https://pubchem.ncbi.nlm.nih.gov/#query=C40H82O2) | 595.1 | 20.311 |  | 22.33 |  |  |  | Anti-inflammatory activity. [13] |
| 49 | Epilupeol | *Triterpenoid* | [C_40_H_82_O_2_](https://pubchem.ncbi.nlm.nih.gov/#query=C40H82O2) | 426.729 | 20.980 |  | 0.30 |  |  |  | Antiviral activity[19] |
| 50 | Tetracontane-1,40-diol |  | [C_40_H_82_O_2_](https://pubchem.ncbi.nlm.nih.gov/#query=C40H82O2) | 595.1 | 21.290 |  | 12.09 |  |  |  | Anti-inflammatory activity. [13] |
| 51 | Methyl 2.alpha.,3.beta.-dihydroxyolean-12-en-28-oate | Triterpenoid | C_31_H_50_O_4_ | 486.726 | 21.705 |  | 3.78 |  |  |  |  |
| 52 | Ursolic aldehyde | Triterpenoid | C_30_H_48_O_2_ | 440.7009 | 22.037 |  | 0.27 |  |  |  | Anticancer. [20] |
| 53 | Urs-12-en-28-oic acid, 3-hydroxy-, methyl ester (3beta) | Triterpenoid | [C_30_H_48_O_3_](https://pubchem.ncbi.nlm.nih.gov/#query=C30H48O3) | 470.3756 | 22.446 |  | 2.88 | 6.34 |  |  |  |
| 54 | Ursolic aldehyde | Triterpenoid | C_30_H_48_O_2_ | 440.7009 | 22.755 |  | 0.99 | 1.07 |  |  | Anticancer. [20] |
| 55 | Uvaol | Triterpene | C_30_H_50_O_2_ | 442.7168 | 23.950 |  | 0.58 | 1.03 |  |  | antibacterial activity.[21] |
| 56 | 4-Vinylbenzene-1,2-diol | Phenol | [C_8_H_8_O_2_](https://pubchem.ncbi.nlm.nih.gov/#query=C8H8O2) | 136.15 | 5.055 |  |  | 0.39 |  |  |  |
| 57 | Methyl 8,11,14-heptadecatrienoate | Fatty acid methyl ester | [C_18_H_30_O_2_](https://pubchem.ncbi.nlm.nih.gov/#query=C18H30O2) | 278.4 | 10.001 |  |  | 0.28 |  |  |  |
| 58 | Tridecanoic acid, 4,8,12-trimethyl-, meth | *Sesquiterpenoid* | [C_16_H_32_O_2_](https://pubchem.ncbi.nlm.nih.gov/#query=C16H32O2) | 256.42 | 10.220 |  |  | 0.60 |  |  |  |
| 59 | l-Norvaline, N-(2-methoxyethoxycarbonyl)-, isohexyl ester | Ester | C_18_H_35_NO_5_ | 345.47 | 10.294 |  |  | 0.37 |  |  |  |
| 60 | Pent-4-enoylamide, 2-methyl-N-(2-butyl)-N-pentyl- | Amide | C1_8_H_35_NO | 281.47 | 11.062 |  |  | 0.28 |  |  |  |
| 61 | 6,9-Octadecadienoic acid, methyl ester | Fatty acid methyl ester | C_19_H_34_O_2_ | 294.5 | 12.130 |  |  | 0.30 |  | 0.78 |  |
| 62 | Octadecanamide | Fatty acid amide | C_18_H_37_NO | 283.5 | 12.390 |  |  | 0.25 |  |  |  |
| 63 | 1,4-Benzenedicarboxylic acid, bis(2-ethyethylhexyl) ester | Pthalate ester | C_24_H_38_O_4_ | 390.56 | 15.136 |  |  | 1.70 |  |  |  |
| 64 | 2,6,10,14-Hexadecatetraen-1-ol, 3,7,11, 15-tetramethyl-, acetate | *Terpenoid* | C_22_H_36_O_2_ | 332.52 | 16.388 |  |  | 0.27 |  |  |  |
| 65 | Hexadecanoic acid, eicosyl ester | Fatty acid ester | C_36_H_72_O_2_ | 536.9557 | 18.756 |  |  | 1.45 |  |  |  |
| 66 | Hexadecanoic acid, tetradecyl ester | Fatty acid ester | C_30_H_60_O_2_ | 452.7962 | 19.015 |  |  | 0.83 |  |  |  |
| 67 | 9-Hexadecenoic acid, 9-octadecenyl ester (Z,Z) | Fatty acid | C_34_H_64_O_2_ | 504.87 | 21.411 |  |  | 1.77 |  |  |  |
| 68 | alpha.-D-Galactopyranose, 2,3,4,6-tetrakis-O-(trimethylsilyl) | Galactose sugar | C_26_H_58_O_6_Si_4_ | 570.20 | 4.195 |  |  |  | 1.26 |  |  |
| 69 | 2-Furancarboxaldehyde, 5[[(trimethylsilyl)oxy]methyl] | furan carboxaldehydes | C_9_H_14_O3Si | 110.1106 | 4.421 |  |  |  | 0.20 |  |  |
| 70 | 2,3-Dimercapto-1-propanol, O,S-bis(trimethylsilyl)- (isomer) | Thiol | C_9_H_22_O_2_SSi_2_. | 250.55 | 5.774 |  |  |  | 0.39 |  |  |
| 71 | .beta.-D-Fructofuranose, 1,2,3,4,6-penkis-O-(trimethylsilyl)- | Furanose | C21H52O6Si5 | 541.1 | 5.878 |  |  |  | 0.17 |  |  |
| 72 | D-(-)-Tagatofuranose, pentakis(trimethylsilyl) ether (isomer) | Furanose | C_21_H_52_O_6_Si_5_ | 541.0615 | 6.046 |  |  |  | 0.83 |  |  |
| 73 | D-(+)-Ribono-1,4-lactone (R,R,R)-, 3TMS Derivative | Lactone | C_14_H_32_O_5_Si_3_ | 364.6574 | 6.191 |  |  |  | 0.18 |  |  |
| 74 | 4-Hydroxy-4,11b-dimethyltetradecahydro-6a,9-methanocyclo. |  | C_14_H_3_0O. | 202.46 | 6.695 |  |  |  | 0.41 |  |  |
| 75 | Lignoceric acid, (TMS derivative) | *Fatty acid* | [C_19_H_46_O_6_Si_4_](https://pubchem.ncbi.nlm.nih.gov/#query=C19H46O6Si4) | 482.9 | 6.863 |  |  |  | 0.80 |  |  |
| 76 | Methyl .alpha.-D-glucofuranoside (TMS derivative) | Glycoside | C_27_H_56_O_2_Si | 440.8178 | 6.863 |  |  |  | 1.38 |  |  |
| 77 | 5-Ethyl-2,2,8,8-tetramethyl-4-propyl-3,7-dioxa-2,8-disilanonane |  | C_11_H_28_O2Si_2_ | 248.51 | 6.976 |  |  |  | 3.24 |  |  |
| 78 | (Z)-3-Hexenyl .beta.-glucopyranoside | Glycoside | [C_12_H_22_O_6_](https://pubchem.ncbi.nlm.nih.gov/#query=C12H22O6) | 262.30 | 7.396 |  |  |  | 1.48 |  |  |
| 79 | Methyl .alpha.-D-glucofuranoside, 4TMS | Glycoside | [C_19_H_46_O_6_Si_4_](https://pubchem.ncbi.nlm.nih.gov/#query=C19H46O6Si4) | 482.9 | 7.560 |  |  |  | 3.88 |  |  |
| 80 | beta.-D-Galactopyranoside, methyl 2,4,6-tris-O-(trimethylsilyl) | Glycoside | [C_18_H_40_O_7_Si_3_](https://pubchem.ncbi.nlm.nih.gov/#query=C18H40O7Si3) | 452.8 | 7.709 |  |  |  | 5.26 |  |  |
| 81 | D-Ribofuranose, 1,2,3,5-tetrakis-O-(trimethylsilyl) | carbohydrate | [C_17_H_42_O_5_Si_4_](https://pubchem.ncbi.nlm.nih.gov/#query=C17H42O5Si4) | 438.9 | 7.779 |  |  |  | 1.69 |  |  |
| 82 | Myristic acid, TMS derivative | Saturated fatty acid | C_17_H_36_O_2_Si | 300.5520 | 7.845 |  |  |  | 3.84 |  |  |
| 83 | beta.-D-Mannopyranoside, methyl 2,3,4,6-tetrakis-O-(trimethylsilyl) | Glycoside | C_6_H_12_O_6_C_16_H_48_Si_4_ | 862.93 | 8.296 |  |  |  | 1.43 |  |  |
| 84 | .beta.-D-Xylopyranose, 4TMS | carbohydrate | C_17_H_42_O_5_Si_4_ | 438.8544 | 8.296 |  |  |  | 1.46 |  |  |
| 85 | 9,12-Octadecadienoic acid (Z,Z)- TMS derivative | Fatty acid | C_21_H_40_O_2_Si | 352.6266 | 10.926 |  |  |  | 0.50 |  |  |
| 86 | 5,8,11-Eicosatrienoic acid, (Z)- TMS derivative | Fatty acid | C_23_H_42_O_2_Si | 378.6639 | 10.971 |  |  |  | 1.50 |  | Anti-inflammatory [22] |
| 87 | Stearic acid TMS derivative | *saturated fatty acid* | C_21_H_44_O_2_Si | 356.6584 | 11.196 |  |  |  | 0.18 | 3.12 |  |
| 88 | Epigallocatechin 6TMS | *Flavan* | C_33_H_62_O_7_Si_6_ | 739.3542 | 16.319 |  |  |  | 0.30 |  |  |
| 89 | Stigmast-5-ene, 3.beta.-(trimethylsiloxy)-, (24S)- | Phytosterol | C_32_H_58_OSi | 486.8878 | 19.351 |  |  |  | 1.31 |  |  |
| 90 | Dammaran-3-one, 20,24-epoxy-25-hydroxy-(20R 24S) | Triterpenoids | C_30_H_50_O_3_ | 458.716 | 20.465 |  |  |  | 0.17 |  |  |
| 91 | Lup-20(29)-en-28-al, 3-(trimethylsilyl)oxy, (3.beta) | Triterpenoids | C_33_H_56_O_2_Si | 512.8820 | 21.301 |  |  |  | 0.96 |  |  |
| 92 | Lup-20(29)-ene, 3,28-bis[(trimethylsilyl)oxy]-, (3.beta.)- | Triterpenoid | C_33_H_56_O_2_Si | 512.8820 | 21.491 |  |  |  | 0.59 |  |  |
| 93 | Glycerol | Polyol | C_12_H_32_O_3_Si_3_ | 308.6372 | 24.193 |  |  |  |  | 4.33 |  |
| 94 | Eicosane | Alkane | C_20_H_42_ | 282.5475 | 6.730 |  |  |  |  | 0.77 |  |
| 95 | 2-Ethyl 1,3-bis(trimethylsilyl) 2-((trimethylsilyl)oxy)propane |  | C_17_H_36_O_7_Si_3_ | 436.72 | 7.276 |  |  |  |  | 3.82 |  |
| 96 | 7,9-Di-tert-butyl-1-oxaspiro(4,5)deca-6,9-diene-2,8-dion | Flavonoid | C_17_H_24_O_3_ | 276.3707 | 8.403 |  |  |  |  | 0.99 | anti-mineralocorticoid. [23] |
| 97 | n-Hexadecanoic acid | Diterpene | C_16_H_32_O_2_ | 256.4241 | 13.755 |  |  |  |  | 1.28 | Anti-inflammatory activity [24] |

1. Sowmya S, Perumal PC, Gopalakrishnan VK. Chromatographic and spectrophotometric analysis of bioactive compounds from Cayratia trifolia (L.) stem. International Journal of Pharmacy and Pharmaceutical Sciences. 2016:56-64.

2. Sankpal MM. DETERMINATION OF PHYTOCONSTITUENTS IN ANNONA RETICULATA LINN. METHANOLIC LEAF EXTRACT USING GCMS. 2022.

3. Amsalem E, Teal P, Grozinger CM, Hefetz A. Precocene-I inhibits juvenile hormone biosynthesis, ovarian activation, aggression and alters sterility signal production in bumble bee (Bombus terrestris) workers. Journal of Experimental Biology. 2014;217(17):3178-85.

4. Patil K, Singh DM. GC-MS Analysis of fresh water Cylindrospermum sp. PCC518, Cylindrospermum sp. PCC 567 ethanol and hexane extracts. Int J Herb Med. 2022;10:15-25.

5. Guan X, Ge D, Li S, Huang K, Liu J, Li F. Chemical composition and antimicrobial activities of Artemisia argyi Lévl. et Vant essential oils extracted by simultaneous distillation-extraction, subcritical extraction and hydrodistillation. Molecules. 2019;24(3):483.

6. Phillips S, Rao MRK, Prabhu K, Priya M, Kalaivani S, Ravi A, et al. Preliminary GC-MS analysis of an Ayurvedic medicine “Kulathadi Kashayam”. J Chem Pharm Res. 2015;7:393-400.

7. Olaoluwa O, Moronkola D, Taiwo O, Iganboh P. Volatile oil composition, antioxidant and antimicrobial properties of Boerhavia erecta L. and Euphorbia hirta L. Trends in Phytochemical Research. 2018;2(3):171-8.

8. Save S, Lokhande R, Chowdhary A. Determination of 1, 2-Benzenedicarboxylic acid, bis (2-ethylhexyl) ester from the twigs of Thevetia peruviana as a Colwell Biomarker. J Innov Pharm Biol Sci. 2015;2(3):349-62.

9. Devi JAI, Muthu AK. GAS CHROMATOGRAPHY-MASS SPECTROMETRY ANALYSIS OF PHYTOCOMPONENTS IN THE ETHANOLIC EXTRACT FROM WHOLE PLANT OF LACTUCA RUNCINATA DC. GAS. 2015;8(1).

10. Choi JM, Lee EO, Lee HJ, Kim KH, Ahn KS, Shim BS, et al. Identification of campesterol from Chrysanthemum coronarium L. and its antiangiogenic activities. Phytotherapy Research. 2007;21(10):954-9.

11. Zhao L, Chen X-J, Zhu J, Xi Y-B, Yang X, Hu L-D, et al. Lanosterol reverses protein aggregation in cataracts. Nature. 2015;523(7562):607-11.

12. Aragao GF, Pinheiro MCC, Bandeira PN, Lemos TLG, Viana GSdB. Analgesic and anti-inflammatory activities of the isomeric mixture of alpha-and beta-amyrin from Protium heptaphyllum (Aubl.) march. Journal of herbal pharmacotherapy. 2008;7(2):31-47.

13. Elangovan M, Dhanarajan M, Elangovan I, Elangovan M. Determination of bioactive compounds from the Petroleum ether leaf extract of Moringa oleifera and Phyllanthus emblica using GC-MS analysis. World Journal of Pharmaceutical Research. 2015;4(3):1284-98.

14. Prakash P, Namasivayam SKR. Screenig of bioactive compounds by GC-Ms from Fusarium venenatum. International Journal of PharmTech Research. 2014;6(6):1833-7.

15. Ujvary I, Christie R, Evans-Brown M, Gallegos A, Jorge R, de Morais J, et al. DARK classics in chemical neuroscience: etonitazene and related benzimidazoles. ACS Chemical Neuroscience. 2021;12(7):1072-92.

16. Dar KB, Parry RA, Bhat AH, Beigh AH, Ahmed M, Khaja UM, et al. Immunomodulatory efficacy of Cousinia thomsonii CB Clarke in ameliorating inflammatory cascade expressions. Journal of Ethnopharmacology. 2023;300:115727.

17. Malathi K, Ramaiah S. Ethyl iso-allocholate from a medicinal rice Karungkavuni inhibits dihydropteroate synthase in Escherichia coli: A molecular docking and dynamics study. Indian Journal of Pharmaceutical Sciences. 2017;78(6):780-8.

18. Idan SA, Al-Marzoqi AH, Hameed IH. Spectral analysis and anti-bacterial activity of methanolic fruit extract of Citrullus colocynthis using gas chromatography-mass spectrometry. African Journal of Biotechnology. 2015;14(46):3131-58.

19. Chowdhury B, Hussaini F, Shoeb A. Antiviral constituents from Vicoa indica. International Journal of Crude Drug Research. 1990;28(2):121-4.

20. Wang W, Zhao C, Jou D, Lü J, Zhang C, Lin L, et al. Ursolic acid inhibits the growth of colon cancer-initiating cells by targeting STAT3. Anticancer research. 2013;33(10):4279-84.

21. Martins A, Vasas A, Viveiros M, Molnár J, Hohmann J, Amaral L. Antibacterial properties of compounds isolated from Carpobrotus edulis. International journal of antimicrobial agents. 2011;37(5):438-44.

22. Hsu L-C, Wen Z-H, Chen H-M, Lin H-T, Chiu C-M, Wu H-C. Evaluation of the anti-inflammatory activities of 5, 8, 11-cis-Eicosatrienoic acid. 2013.

23. Godara P, Dulara BK, Barwer N, Chaudhary NS. Comparative GC-MS Analysis of Bioactive Phytochemicals from Different Plant Parts and Callus of Leptadenia reticulata Wight and Arn. Pharmacognosy Journal. 2019;11(1).

24. Aparna V, Dileep KV, Mandal PK, Karthe P, Sadasivan C, Haridas M. Anti‐inflammatory property of n‐hexadecanoic acid: structural evidence and kinetic assessment. Chemical biology & drug design. 2012;80(3):434-9.
