# Supplementary material for: Hoslundia opposita vahl; a potential source of bioactive compounds with antioxidant and antibiofilm activity for wound healing
Source: BMC Complement Med Ther. 2024 Jun 17;24:236. doi: 10.1186/s12906-024-04540-z (PMC11181642; doi:10.1186/s12906-024-04540-z)
Supplement: Supplementary file 2 — Supplementary Material 2 [file 12906_2024_4540_MOESM2_ESM.docx]

**ADDITIONAL FILE TWO**

**Table2:** **Phytochemicals screened from the methanol extract of H. opposita Vahl in MRM mode by LC-MS.**

| **NO** | **Tentative Name** | **Precursor ion;**  **[M]^+^, [M+H]^+^ or [M-H]^-^** | **Product ion** | **CE (V)** | **Polarity** | **Class** |
| --- | --- | --- | --- | --- | --- | --- |
| 1 | Saikosaponin C | 927.0 | 511, 421 | 25, 45 | Positive | Saponin |
| 2 | Saikosaponin D | 781.0 | 617, 331 | 25, 45 | Positive | Saponin |
| 3 | Saikosaponin A | 781.0 | 617, 331 | 15, 35 | Positive | Saponin |
| 4 | Hesperidin | 611.2 | 449, 303 | 10, 20 | Positive | Flavonone/flavonoid |
| 5 | Neohesperidin | 611.2 | 303, 153 | 20, 40 | Positive | Flavonone/flavonoid |
| 6 | 8-O-demethyl dihydrochelerythrine-9-glucoside | 498.0 | 336, 250 | 20, 40 | Positive | Alkaloid |
| 7 | Magnocurarine-6-glucoside | 476.0 | 314, 107 | 20, 40 | Positive | Alkaloid |
| 8 | 7-methoxy-2- methylhigenamine-4'-glucoside | 462.0 | 300, 107 | 20, 40 | Positive | Alkaloid |
| 9 | 4'-methoxy-ribalinine-3'-glucoside | 452.0 | 290, 218 | 20, 40 | Positive | Alkaloid |
| 10 | Lineaflavone A | 433.2 | 401, 223 | 20, 40 | Positive | Flavone/flavonoid |
| 11 | Ribalinine- 3'-glucoside | 422.0 | 260, 188 | 20, 40 | Positive | Others |
| 12 | Lineaflavone B/3-o-benzoylhosloppone | 419.2 | 401, 281 | 20, 40 | Positive | Flavone/flavonoid |
| 13 | Diacetoxy- 8-gingerdiol | 409.3 | 349, 137 | 40, 40 | Positive | Others |
| 14 | Lineaflavone D | 401.2 | 369, 223 | 15, 35 | Positive | Flavone/flavonoid |
| 15 | Lineaflavone C | 387.2 | 369, 223 | 20, 40 | Positive | Flavone/flavonoid |
| 16 | Diacetoxy- 6-gingerdiol | 381.2 | 321, 137 | 35, 40 | Positive | Others |
| 17 | 8-Acetyl obovatin | 381.0 | 339, 217 | 20, 35 | Positive | Prenylated flavonoid |
| 18 | 8-methoxy- dihydronitidine | 380.0 | 364 | 20 | Positive | Alkaloid |
| 19 | 6-Hydroxy-10-methylsanguinarine | 380.0 | 362, 275 | 40, 40 | Positive | Alkaloid |
| 20 | 8-methoxy-dihydronitidine | 380.0 | 249 | 40 | Positive | Alkaloid |
| 21 | 5-hydroxy- 7-methoxysaniculamin | 371.1 | 353, 251 | 25, 35 | Positive | Alkaloid |
| 22 | a-allocryprotopine | 370.1 | 352, 206 | 20, 40 | Positive | Dibenzenzic alkaloid |
| 23 | N-methyl tetrahydropalmitine | 370.0 | 308, 145 | 20, 40 | Positive | Alkaloid |
| 24 | Dimethyl jatrorrhizine | 368.0 | 352, 278 | 20, 40 | Positive | Isoquinoline alkaloid |
| 25 | 6-methyl- 5,6-dihydrofagaridine | 366.0 | 350, 276 | 40 | Positive | Alkaloid |
| 26 | Dihydrochelerubine | 364.0 | 349, 319 | 68, 70 | Positive | Alkaloid |
| 27 | Oxychelerythrine | 364.0 | 348, 276 | 70, 75 | Positive | Alkaloid |
| 28 | 8-methoxyisotembetarine | 358.0 | 298, 105 | 20, 40 | Positive | Alkaloid |
| 29 | N-methyltetra hydrocolumbamine | 356.2 | 192, 149 | 20, 40 | Positive | Alkaloid |
| 30 | Hunnemannine | 356.1 | 338, 149 | 20, 40 | Positive | Alkaloid |
| 31 | Tetrahydropalmatine | 356.0 | 340, 149 | 20, 40 | Positive | Alkaloid |
| 32 | Xanthoplanine | 356.0 | 311, 177 | 20, 40 | Positive | Alkaloid |
| 33 | Menisperine | 356.0 | 311, 177 | 20, 40 | Positive | Alkaloid |
| 34 | 2-dimethoxy- 3-hydroxybuegenine | 354.0 | 339, 252 | 20, 40 | Positive | Alkaloid |
| 35 | N-methylcanadine | 354.0 | 338, 149 | 20, 40 | Positive | Alkaloid |
| 36 | Buegenine | 352.0 | 337, 266 | 20, 40 | Positive | Alkaloid |
| 37 | Palmatine | 352.0 | 336, 264 | 20, 40 | Positive | Alkaloid |
| 38 | 10-Gingerol | 351.3 | 333, 115 | 35, 40 | Positive | Others |
| 39 | Fagorinine | 351.1 | 335, 236 | 20, 40 | Positive | Alkaloid |
| 40 | Dihydrochelerythrine | 350.0 | 335, 290 | 20, 40 | Positive | Alkaloid |
| 41 | Chelerythrine | 348.4 | 317, 290 | 80, 85 | Positive | Alkaloid |
| 42 | Nitidine | 348.0 | 333, 290 | 20, 40 | Positive | Alkaloid |
| 43 | 5,3',4'-trihydroxy-3-methoxy- 6,7-methylene dioxyflavone | 345.0 | 329, 297 | 35, 40 | Positive | Flavone/flavonoid |
| 44 | (-)-Xylopinidine | 344.0 | 301, 137 | 20, 40 | Positive | Alkaloid |
| 45 | N-trans- Feruloyl-3-methoxytyramine | 344.0 | 178, 147 | 35,40 | Positive | Alkaloid |
| 46 | Magnoflorine | 342.0 | 297, 191 | 20, 40 | Positive | Alkaloid |
| 47 | Laurifoline | 342.0 | 282, 191 | 20, 40 | Positive | Alkaloid |
| 48 | 2-methoxy-5-dehydroscoulerine | 340.0 | 325, 119 | 20, 40 | Positive | Alkaloid |
| 49 | Atrorrhizine | 338.0 | 322, 265 | 20, 40 | Positive | Alkaloid |
| 50 | Acetoxy-6-gingerol | 337.2 | 319, 137 | 35, 40 | Positive | Others |
| 51 | Atalantoflavone | 337.1 | 321, 161 | 10, 35 | Positive | Flavone/flavonoid |
| 52 | p- Coumaroyl quinic acid | 337.1 | 191, 111 | 35, 40 | Negative | Hydroxycinnamic acid derivative/phenolic acid |
| 53 | Berberine | 336.1 | 320, 318 | 40, 40 | Positive | Alkaloid |
| 54 | 8-O-demethyldihydrochelerythrine | 336.0 | 321, 250 | 60, 75 | Positive | Alkaloid |
| 55 | Dihydrosanguinarine | 334.1 | 319, 276 | 72, 75 | Positive | Alkaloid |
| 56 | 8-O-demethylcheerythrine | 334.1 | 319, 264 | 20, 40 | Positive | Alkaloid |
| 57 | Sanguinarine | 332.1 | 317, 274 | 40, 40 | Positive | Alkaloid |
| 58 | 3,7-trimethylquercetin | 331.1 | 314, 265 | 35, 40 | Positive | Flavonol/flavonoid |
| 59 | Reticuline | 330.0 | 207, 115 | 20, 40 | Positive | Alkaloid |
| 60 | Avicine | 328.1 | 283, 121 | 20, 40 | Positive | Alkaloid |
| 61 | 8-Gingerol | 323.2 | 305, 115 | 35, 40 | Positive | Others |
| 62 | Berberubine | 322.1 | 307, 264 | 50, 60 | Positive | Alkaloid |
| 63 | 9,10-didemethylchelerythrine | 320.1 | 305, 248 | 60, 75 | Positive | Alkaloid |
| 64 | Erylivingstone | 317.1 | 299, 257 | 25, 35 | Positive | Alkaloid |
| 65 | Tamarixetin | 317.1 | 301, 239 | 35, 40 | Positive | Alkaloid |
| 66 | Hosloppone | 315.1 | 300, 231 | 10, 35 | Positive | Flavonone |
| 67 | Magnocurarine | 314.1 | 269, 107 | 20, 40 | Positive | Alkaloid |
| 68 | N-trans-Feruloyl-tyramine | 314.0 | 178, 147 | 35, 40 | Positive | Alkaloid of hydroxycinnamic acid |
| 69 | Methyl-6-gingerol | 309.1 | 291, 151 | 35, 40 | Positive | Others |
| 70 | Quercetin | 303.1 | 285, 201 | 25, 30 | Positive | Flavonol/Flavonoid |
| 71 | 7-methoxy-N-methylhigenamine | 300.0 | 269, 107 | 20, 40 | Positive | Alkaloid |
| 72 | 6-Gingerdiol | 297.2 | 261, 137 | 35, 40 | Positive | Others |
| 73 | 6-Gingerol | 295.2 | 277, 115 | 35, 40 | Positive | Others (β-hydroxy ketones) |
| 74 | 1-dehydro- 6-gingerdione | 291.2 | 177, 149 | 35, 40 | Positive | Others |
| 75 | 4'-methoxy-ribalinine | 290.1 | 272, 132 | 20, 40 | Positive | Alkaloid |
| 76 | Luteolin | 287.1 | 259, 153 | 15, 25 | Positive | Flavone/flavonoid |
| 77 | Geraldone | 285.1 | 253, 137 | 20, 40 | Positive | Flavonone |
| 78 | Coumaroyl-tyramine | 284.1 | 149, 120 | 20, 40 | Positive | Hydroxybenzoic acid derivative |
| 79 | 1,2-dihydroxy- 3-methoxy- 10-methyl- 9-acridone | 272.1 | 257, 135 | 20, 40 | Positive | Acridines |
| 80 | Apigenin | 271.1 | 259, 153 | 10, 35 | Positive | Flavone |
| 81 | Gamma- glutamyl-S- methylcysteine | 265.1 | 145, 139 | 35, 40 | Positive | Amino acid |
| 82 | Skimmianine | 260.1 | 245, 202 | 25, 40 | Positive | Alkaloid |
| 83 | Ribalinine | 260.1 | 242, 134 | 20, 40 | Positive | Alkaloid |
| 84 | Chrysophanol | 255.1 | 237, 181 | 25, 40 | Positive | Trihydroxyanthraquinone |
| 85 | Haplopine | 246.1 | 231, 156 | 20, 40 | Positive | Quinoline alkaloid |
| 86 | Propoxy- methoxy coumarin | 233.1 | 215 | 35 | Positive | Coumarin |
| 87 | Thalifoline | 208.1 | 176, 115 | 25, 30 | Positive | Alkaloid |
| 88 | Edulitine | 206.1 | 190, 132 | 25, 30 | Positive | Quinoline/ alkaloid |
| 89 | Limettin | 206.0 | 178, 73 | 35, 40 | Positive | Coumarins |
| 90 | 3,4-dihydro-6,7-dimethoxy- isoquinoline | 192.1 | 177, 133 | 15, 20 | Positive | Alkaloid |
| 91 | Psoralen | 187.1 | 131, 115 | 33, 40 | Positive | Furanocoumarin |
| 92 | Esculetin | 179.1 | 123, 177 | 27, 40 | Positive | Coumarin |
| 93 | Vanillic acid | 167.1 | 152, 123 | 35, 40 | Positive | Hydroxycinnamic acid derivative/phenolic acid |
| 94 | p- Coumaric acid | 164.1 | 163, 151 | 35, 40 | Positive | Hydroxycinnamic acid derivative/phenolic acid |
| 95 | Allicine | 163.1 | 87, 73 | 35, 40 | Positive | diallyl thiosulfinate /organosulpur |
| 96 | Des-N- methyl- thalifoline | 147.1 | 103, 91 | 20, 40 | Positive | Alkaloid |
| 97 | Coumarin | 147.0 | 177, 89 | 20, 40 | Positive | Coumarin |
|  |  |  |  |  |  |  |
| 98 | (+)-syringaresinolbeta-D-glucoside/3-o-benzoyl hosloquinone | 579.1 | 417, 387 | 35, 40 | Negative | Lignan |
| 99 | Limocitrol- O-glucoside (flavonoid-3-o-glycoside) | 537.1 | 522, 359 | 35, 40 | Negative | Flavonoid |
| 100 | Caffeic acid- hexoside- O-pentoside | 473.0 | 341, 341 | 35, 40 | Negative | Hydroxycinnamic acid derivative/phenolic acid |
| 101 | Limonin(limonoic acid di-delta-lactone) | 469.1 | 455, 191 | 35, 40 | Negative | limonoid, |
| 102 | Scoparin | 461.2 | 371, 311 | 35, 40 | Negative | Coumarin |
| 103 | Isoorientin(luteolin-6-C-glucoside) | 447.1 | 297, 429 | 35, 40 | Negative | Flavone/flavonoid |
| 104 | Vitexin | 431.1 | 311, 341 | 35, 40 | Negative | Flavone/flavonoid |
| 105 | Feruloyl quinic acid | 367.1 | 193, 173 | 35, 40 | Negative | Hydroxycinnamic acid derivative/phenolic acid |
| 106 | Limocitrin | 345.1 | 345, 330 | 35, 40 | Negative | Coumarin |
| 107 | dihydro-caffeoyl-O- glucoside | 343.1 | 181, 137 | 35, 40 | Negative | Hydroxycinnamic acid derivative |
| 108 | Tetremethoxy flavone | 341.1 | 326, 255 | 35, 40 | Negative | Flavone |
| 109 | Eriodictyol | 287.1 | 151,107 | 35, 40 | Negative | Flavonone/flavonoid |
| 110 | Propoxy-methoxy coumarin | 233.1 | 111 | 35, 40 | Negative | Coumarin |
| 111 | Syringic acid | 197.1 | 182, 153 | 35, 40 | Negative | Hydroxycinnamic acid derivative/phenolic acid |
| 112 | Ferulic acid | 193.1 | 178, 134 | 35, 40 | Negative | Hydroxycinnamic acid derivative/phenolic acid |
| 113 | Citric acid | 191.1 | 173, 87 | 35, 40 | Negative | Carboxylic acid |
| 114 | Isocitric acid | 191.1 | 173, 85 | 35, 40 | Negative | Carboxylic acid |
| 115 | Malic acid | 133.1 | 115, 71 | 35, 40 | Negative | Carboxylic acid |

**Table 3: Phytochemicals screened from the methanol extract of H. opposita Vahl in scan mode by LC-MS**

| **No, RT** | **Tentative identification** | **(M-H)^-^(*m/z*)** | **(M+H)^+^, (M+2H)^+^, [M+Na]^+^(*m/z*)** | ***m/z* of MS/MS for -ve ion mode (abundance)** | ***m/z* of MS/MS for +ve ion mode (abundance)** | **Class** |
| --- | --- | --- | --- | --- | --- | --- |
| Cpd 1: 0.245 | Quercetin | 300.7 |  | 151, 179 |  | Flavonol/Flavonoid |
| Cpd 112: 0.255 | Ferulic acid | 193.1 |  | 134, 103 |  | Hydroxycinnamic acid |
| Cpd 112:: 0.255 | Ferulic acid | 193.1 |  | 134, 103 |  | Hydroxycinnamic acid |
| Cpd 4: 0.272 | p-coumaric acid | 163.0 |  | 119, 93 |  | Hydroxycinnamic acid |
| Cpd 5: 0.265 | Gallic acid | 169.1 |  | 79, 125 |  | Hydroxybenzoic acid |
| Cpd 6: 0.372 | Neohesperidin |  | 611.2 |  | 85, 153, 303 | Flavonone/flavonoid |
| Cpd 7: 0.8 | Rutin |  | 611.2 |  | 85, 153, 303 | Flavonol/Flavonoid |
| Cpd 8: 1.274 | 2-Methoxycinnamic acid |  | 144.1 |  | 144, 104, 118 | Hydroxycinnamic acid derivative |
| Cpd 9: 1.282 | Sinapoyl malate | 341.1 |  | 128, 188, 126, 197, 255 |  | Hydroxycinnamic acid derivative |
| Cpd 10: 1.386 | 2-Methoxycinnamic acid derivative |  | 225.9 |  | 144, 114 | Hydroxycinnamic acid derivative |
| Cpd 11: 1.393 | Caffeic acid | 179.0 |  | 134, 135 |  | Hydroxycinnamic acid |
| Cpd 12: 18.153 | 2-methoxy-5-dehydro-scoulerine |  | 362.2^*Na^ |  | 340, 114, 163, 227 | Alkaloid |
| Cpd 13: 18.161 | Syringic acid hexoside | 359.0 |  | 161, 197 |  | Hydroxycinnamic acid derivative |
| Cpd 14: 18.646 | 2-methoxy-5-dehydro-scoulerine isomer |  | 362.2^*Na^ |  | 340, 114, 163, 227 | Alkaloid |
| Cpd 15: 18.654 | Syringic acid hexoside derivatives (methyl syringate hexoside) | 374.2 |  | **338**, 113, 359 |  | Hydroxycinnamic acid derivative |
| Cpd 16: 19.204 | Unknown |  | 453.3 |  | 197, 114 |  |
| Cpd 17: 19.211 | Neodiosmin | 609.2 |  | 487, 144, 563, 231 |  | Flavone/flavonoid |
| Cpd 18: 19.442 | Unknown |  | 453.0 |  | 453, 322, 227, 114 |  |
| Cpd 19: 19.315 | Eriodictyol-7-O-glucuronide | 462.8 |  | 593, 463, 231, 193 |  | Flavonone/flavonoid |
| Cpd 20: 20.095 | Glycyrrhizin |  | 565.1 |  | 523, 452, 322, 227, 409, 171, 114 | Saponin |
| Cpd 20: 20.095 | Scoparin (8-glucosyl-4¢,5,7-trihydroxy-3¢-methoxyflavone) | 462.9 |  | 113 |  | Flavone/flavonoid |
| Cpd 21: 20.239 | Dehydrated isovitex |  | 413.1 |  | 322, 327, 366, 395, 409, 413, 473, 181, 114 | Flavone/flavonoid |
| Cpd 21: 20.239 | Scoparin (8-glycosyl-4¢,5,7-trihydroxy-3¢-methoxyflavone) isomer | 462.9 |  | 463, 281, 113 |  | Flavone/flavonoid |
| Cpd 22: 20.509 | Hoslundin 1 |  | 408.1 |  | 366, 408, 308, 181, | Favonoid |
| Cpd 22: 20.509 | Diosmin | 609.1 |  | 463, 325, 364, 293 |  | Flavone/flavonoid |
| Cpd 23: 20.605 | Neo 5-0-Methylhoslundin |  | 423.1 |  | 380, 285, 399, | Isoflavonoid |
| Cpd 23: 20.605 | 5-0-Methylhoslundin | 421 |  | 325 |  | Isoflavonoid |
| Cpd 24: 20.700 | Neo 5-0-Methylhoslundin  isomer |  | 423.1 |  | 407, 423, 114 | Isoflavonoid |
| Cpd 24: 20.700 | 5-0-Methylhoslundin isomer | 421.0 |  | 325, 389, 383, 113 |  | Isoflavonoid |
| Cpd 25: 20.828 | Hosloppin |  | 393.1 |  | 393, 381, 408 | Flavonoid |
| Cpd 26: 20.828 | Hoslundin | 459.0 |  | 325, 381, 311, 113 |  | Isoflavonoid |
| Cpd 27: 20.923 | 3-O-benzoyl hinokiol |  | 407.2 |  | 407, 393, 421, 334 | Flavonoid |
| Cpd 28: 20.923 | Barlerine | 619.1 or 505.1 |  | 505, 325, 227, 487, 113 |  | Alkaloid |
| Cpd 29: 21.242 | Unidentified |  | 507.3 |  | 356, 423, 177, 341 | alkaloid |
| Cpd 30: 21.242 | Barlerine isomer | 487.2 |  | 379, 325, 295, 196, 341 |  | Alkaloid |
| Cpd 31: 21.401 | Saikosaponin D |  | 783.2 |  | 422, 358, 298, 227, 144, 308, 322 | Flavonoid |
| Cpd 32: 21.401 | Esculin sesquihydrate | 339.2 |  | 325, 253, 113, 353 |  | coumarin glucoside/Coumarin |
| Cpd 33: 21.608 | unknown |  | 489.1 |  | 358, 408, 274, 185, 149 |  |
| Cpd 34: 21.608 | Esculin sesquihydrate  isomer | 339.1 |  | 339, 325, 253 |  | coumarin glucoside/Coumarin |
| Cpd 35: 21.751 | Saikosaponin A |  | 783.2 |  | 422, 307, 274, 161, 344 |  |
| Cpd 36: 21.751 | Esculin sesquihydrate  isomer | 339.2 |  | 325, 253, 113 |  | coumarin glucoside/Coumarin |
| Cpd 37: 21.847 | Unknown |  | 542.0 |  | 358, 422, 490, 307, 199, 149, 111 | alkaloid |
| Cpd 38: 21.847 | Unknown | 540.0 |  | 339, 279, 518 |  | alkaloid |
| Cpd 39: 22.054 | Methyl-6-gingerol |  | 308.2 |  | 161 | Others (β-hydroxy ketones) |
| Cpd 40: 22.054 | Esculin sesquihydrate  isomer | 339.2 |  | 339, 255, 113, 277 |  | coumarin glucoside/Coumarin |
| Cpd 41: 22.261 | Unknown |  | 895.5 |  | 843, 688, 325, 455, 424, 393, 283, 149 |  |
| Cpd 42: 22.261 | Unknown | 661.4 |  | 255, 519, 451, 279 |  |  |
| Cpd 43: 22.436 | Digalloylated procyanidin dimer type A |  | 880.8 |  | 325, 843, 424, 640, 387, 114 | Flavan-3-ol/ flavonoid |
| Cpd 44: 22.436 | Eriodictyol-7-O-glucuronide | 465.3 |  | 455, 255, 339, |  | Flavonone/flavonoid |
| Cpd 45: 22.723 | Digalloylated procyanidin dimer type A isomer |  | 880.8 |  | 325, 640, 843, 325, 867 | Flavan-3-ol/ flavonoid |
| Cpd 46: 22.723 | Unknown | 861.6 |  | 255, 455, 465, 281, 405, 113, 762, 519 |  |  |
| Cpd 47: 22.818 | Digalloylated procyanidin dimer type A isomer |  | 880.9 |  | 843, 793, 593, 424, 309, 325 | Flavan-3-ol/ flavonoid |
| Cpd 48: 22.818 | Un identified | 689.6 |  | 519, 451, 405, 283, 453, 465 |  |  |
| Cpd 49: 23.057 | Digalloylated procyanidin dimer type A isomer |  | 880.9 |  | 843, 793, 593, 424, 309, 325 | Flavan-3-ol/ flavonoid |
| Cpd 50: 23.057 | Unknown | 689.6 |  | 519, 451, 405, 283, 453, 465 |  |  |
| Cpd 51: 23.264 | Digalloylated procyanidin dimer type A isomer |  | 880.9 |  | 843, 793, 593, 424, 309, 325 | Flavan-3-ol/ flavonoid |
| Cpd 52: 23.264 | Unknown | 689.6 |  | 519, 451, 405, 283, 453, 465 |  |  |
| Cpd 53: 23.391 | Digalloylated procyanidin dimer type A isomer |  | 880.9 |  | 843, 793, 593, 424, 309, 325 | Flavan-3-ol/ flavonoid |
| Cpd 54: 23.391 | Unknown | 689.6 |  | 519, 451, 405, 283, 453, 465 |  |  |
| Cpd 55: 23.710 | Digalloylated procyanidin dimer type A isomer |  | 880.9 |  | 843, 793, 593, 424, 309, 325 | Flavan-3-ol/ flavonoid |
| Cpd 55: 23.710 | E-3,4,5'-trihydroxy-3'-glucopyranosyl stilbene | 761.6 |  | 565, 451, 311, 405, 465 |  | Stilbenoid |
| Cpd 56: 23.933 | Glycyrrhizic acid ammonium salt |  | 842.8 |  | 793, 463, 338, 114 | Saponin |
| Cpd 57: 23.933 | Eriodictyol-7-O-glucuronide | 465.2 |  | 451, 405 |  | Flavonone/flavonoid |

^*Na^=**[M+Na]^+^**

**Details of LC-MS ANALYSIS**

**Analysis of carboxylic acids**

Three carboxylic acids could be identified i.e. compounds 113, 114 and 115. Fragmentation of the carboxylic acids occurred by release of one or two water (18Da) and carbondioxide (44Da) molecules or both (62Da) and characterized by the ion fragments [M-H-18]^-^ and CO_2_, [M-H-44]^-^, or [M-H-62]^-^. Peak 113, 114, 115 were identified as citric acid, isocitric acid and malic acid respectively [1-3].

**Analysis of hydroxycinnamic acids**

In the MRM study mode, eleven hydroxycinnamic acid and derivatives were tentatively identified (compounds 11, 52, 68, 93, 94, 100, 105, 107, 111 and 112, 116). Cinnamic acids in their free form were detected. p-coumaric acid (94), caffeic acid (11) and syringic acid (111), which were identified using literature [4]. P-coumaryl quinic acid, compound 52, was identified with precursor ion *m/z* 337 with MS/MS fragments 191[quinic -H]^-^ [5]. Using the literature, syringic acid hexosides; [M-H]-, *m/z* 359, identified, showing a typical major fragment; 197 [syringic acid -H]^-^, 329 [M –H- CH_3_]^-^, and 161 [M–H-syringic acid]^-^  [4] (see Figure 12 C in the main text). Ferulic acid was identified at peak 112 with precursor ion, [M - H]^-^ ion at *m/z* 193 and MS^2^ fragments *m/z* 178 [Ferouly- CH_3_]^-^ and 134 [Ferouly-CO_2_-CH_3_]^-^. Compound 105 was identified as that of feruloyl quinic acid having [M - H]^-^ ion at *m/z* 367and MS^2^ fragments *m/z* 193 [ferulic -H]^-^, 173 [quinic acid - H-H_2_O]^-^. Compound 68, was identified as N-trans-feruloyl-tyramine with [M+H]^+^ ion at *m/z* 314 and *m/z* 178 [M+H- tyramine]^+^, 147 [M+H- tyramine-2CH_3_]^+^. Caffeic acid was identified in negative ion mode with [M-H]^-^ ion, *m/z* 179 and MS^2^ fragments *m/z* 135 [M –H-CO_2_]^-^ (as illustrated in the fragmentation pattern in the main text, Figure 12 B) and Caffeic acid- hexoside- O-pentoside was identified with precursor ion 473 with fragment 341 ([M-H]^-^ -132), 312 ([M-H]^-^ -162)[6] Dihydro-caffeoyl-O- glucoside with deprotonated ion *m/z* 343 and fragments *m/z* 181 ([M-H]^-^ -glc.) and 137 ([M-H]^-^ -glc.- CO_2_), consistent with earlier literature [7].

**2.0 Analysis of hydroxybenzoic acids/ other compounds (phenolic acids)**

Compound 93 had [M-H]^-^ ion at *m/z* 167 and had a base peak ion in the MS^2^ spectrum at *m/z* 123 [M-H-CO_2_]^-^, as well as fragments at *m/z* 152 [M-H-CH_3_]^-^ and *m/z* 108 [M-H-CO_2_-CH_3_]^-^ [8]. Gingerols were identified by the protonated molecular ions ([M+H]^+^), H_2_O subtracted protonated molecular ion ([M-H_2_O + H]^+^). Compound 61 and 73 are beta-hydroxy ketones, named as 6-gingerol and 8-gingerol respectively. Their targeted ions were 277 ([M-H_2_O+H]^+^), and 305 ([M-H_2_O+H]^+^), or 277 ([M-H_2_O-CH_3_+H]^+^) for methyl-6-gingerol in agreement with earlier report [9] (see figure 12 D).The MS^1^ ions was *m/z* 295 and 323 respectively producing common fragment ion *m/z* 115 [C_7_H_13_O]^+^ in MS^2^ formed as a result of inductive cleavage of the side chain. Compound 38 was identified as 10-gingerol, Compound 50 was identified as acetoxy-6-gingerol with MS^2^ fragments 319 due to losses of H_2_O from the parent ion. The ion fragment at *m/z* 137 was due to cleavage of alkyl chain with its acetoxy group [10]. The formation of fragment ions at *m/z* 321, [M+H-AcOH]^+^, suggests di-acetylated derivative of 6-gingerdiol thus compound 16 was identified as diacetoxy-6-gingerdiol [10]. Compound 13 with some similarity in fragmentation pattern showed an increase of CH_2_–CH_2_ (28 Da) for adduct of [M+H-AcOH]^+^ to diacetoxy-6-gingerdiol thus identified as diacetoxy- 8-gingerdiol (Jolad et al., 2004). Gingerdione compound showed fragment ions of [M+H]^+^ and [M+H-CH_3_(CH_2_)_n_OCHCH_2_, n = 4]^+^ by CH_2_–CH_2_ (28 Da) increments thus compound 74 is tentatively identified as 1-dehydro-6-gingerdione [11].

**Analysis of lignans**

Compound 98 was identified as syringaresinol β-D-glucoside, showed a deprotonated molecular ion at *m/z* 579 and a major MS/MS fragments 417 attributed to the loss of hexoside moiety [12].

**Analysis of saponins**

The different fragmentation transition pairs of saikosaponin A, SSa, saikosaponin C SSc and saikosaponin D, SSd are: SSa and SSd [M + H]^+^ *m/z*  781 617 [M -glu -H]^+^, 331 [M- aglycone + Na]^+^, SSc [M + H]^+^ *m/z*  927 421 [M -2glu – rha- 2H_2_O + H]^+^ , 511 [M- aglycone + Na]^+^. The fragmentation of saikosaponin A show initial loss of the glucose to generate m/z 617 and successive second loss of another sugar to give 307Da. Retro-Diels-Alder (RDA) cleavage indicated presence of 423 Da and following by loss of one methyl group, *m/z* 3444 is formed (as illustrated in the fragmentation pattern in Figure 12 G main text). This is similar to that of saikosaponin D which were identified in both MRM and scan modes

**3.0 Analysis of flavanones, flavones, flavonols, isoflavonoids and favan-3-ols**

Flavanones, flavones, flavonols, and favan-3-ols were the most identified flavonoids. The fragmentation pathways of flavonoids were followed by Retro-Diels-Alder (RDA). Flavonone give with MS^2^ fragment ions 151[^1,3^A^-^], /153 [^1,3^A^+^], (RDA cleavages of the C-ring of the aglycone) and 161[^0,4^B^+^], / 163 [^0,4^B^+^], (bonds 0 and 4 refer to the O—C-1 and C-4—C-3 bonds of the C-ring) which are characteristic of flavanone fragmentation scheme [13]. In this study, a total of about 30 flavonoids, were identified. Hosloppin, identified in scan mode and showed a protonated molecular ion at *m/z* 393 and fragments 381 after loss of a methylene, as earlier identified from this plant [14]. Hoslondin was detected at *m/z* 379 (see fragmentation in Figure 12 E in main text) consistent with earlier studies on this plant and its methyl derivative was identified as 5-0-methylhoslundin with precursor ion at *m/z* 408 consistent with earlier studies on this plant [15]. Compound 104 was identified as vitex with [M-H]^-^; *m/z* 431 and MS^2^ fragments 341 ([M-H]^-^ - 90), 311 ([M-H]^-^ - 120) [16]. Dehydrated isovitex could be identified in scan mode with fragments 413 ([M-H]^-^ -18). Compound 118 was identified as Tetramethoxy flavone based on comparison of its MS/MS fragmentation pattern with the previously reported data [17]. Diosmin was identified with *m/z* 607 [16]. Chrysoeriol-8-C-glucoside (scoparin) identified in scan mode with earlier reported flavonoid fragments belonging to a flavone [18]. The fragmentation pattern of scoparin is illustrated in Figure 12 G). Compounds 58 and 70 were flavonol compounds. Peak 58 and 70 were respectively identified as 3,7-trimethylquercetin and quercetin after displaying [M-H]^-^; *m/z* 331 and MS^2^ fragments *m/z* 314 (after addition of 14Da, belonging to a methyl on the quercetin aglycon (301Da) and RDA fragment 265, while for the quercetin aglycone were 285 and 201 (the fragmentation of quercetin in positive ion mode is illustrated in Figure 12 F). Flavanones give weak RDA fragments and commonly lose ring B by breaking at different positions [13]. Eriodictyol exhibited [M-H]^-^; *m/z* 287 and MS^2^ main fragments 151 [^1,3^A^-^], formed through retrocyclization RDA cleavages of the C-ring of the aglycone involving 1 and 3 bonds (bonds 1 and 3 refer to the O—C-2 and C-3—C-4 bonds of the C-ring) [13]. which is consistent with that of the aglycone eriodictyol. Conjugation with gluconuride gives eriodictyol-7-O-glucuronide (Farag et al., 2016). Hesperidin,a bioflavonoid was identified in the positive ion mode with protonated ion[M+H]^+^; *m/z* 611 and major MS^2^ fragment at *m/z* 303 [M+H-rutinoside]^+^. Compound 106 was identified as limocitrin in accordance with earlier report [7].

**Analysis of Coumarins**

Coumarins could either be methoxyl, propxyl or acetyl derivatives through the addition of one of 31 units (methoxy), 59 (propoxyl) or 43 mass units respectively to *m/z* 145/147 for coumarin [18]. The [M-H]^-^ ion at *m/z* 175.1 (peak 35) with MS^2^ of characteristic ion *m/z* 131 [M-H-CO_2_]^-^ was identified to be that of the simple coumarin, and esculetin [19]. Compound 86 was identified as propoxy- methoxy coumarin.

**Analysis of Alkaloids**

As noted earlier, alkaloids were the most identified compounds from this plant in this study. In positive ion mode, berberine, palmatine and jatrorrhizine yielded the molecular ion [M]^+^ and the fragment ions [M- ^•^CH_3_]^+•^, [M-^•^ CH_3_- ^•^H]^+^, [M- ^•^CH_3_- ^•^H-CO]^+^ and [M- ^•^CH_3_- ^•^H- 2^•^H]^+^ in MS^2^ as earlier reported [20]. Compound 53 showed a molecular ion [M]^+^ at *m/z* 336, which fragmented to [M-^•^CH_3_-^•^H]^+^ at *m/z* 320 and [M-^•^CH_3_-^•^H-2^•^H]^+^ at *m/z* 318. This compound was tentatively identified as berberine in agreement with earlier literature [20]. Palmatine showed MS^1^ at *m/z* 352 and MS^2^ at *m/z* 336[M-^•^ CH_3_- ^•^H]^+^, 264[M- 2CO_2_]^+^. Compound 31 was identified as tetrahydropalmatine with M]^+^ at *m/z* 356 and MS^2^ at *m/z* 340[M-^•^ CH_3_- ^•^H + 4H]^+^ and perhaps RDA cleavage ion of the ring at *m/z* 149. Compound 24 was identified as dimethyl jatrorrhizine with M^+^ at *m/z* 368 for MS^1^ with MS^2^ at *m/z* 352[M- ^•^CH_3_- ^•^H]^+^ and 278 [M- 2^•^CH_3_- ^•^H-CO)]^+^. Compound 48 was considered to possess a tetrahydroprotoberberine alkaloid skeleton and thus tentatively identified as 2-methoxy- 5-dehydroscoulerine. It generated a protonated molecular ions [M+H]^+^ at *m/z* 340 [20] and MS^2^ fragment ions 325 [M+H-H_2_O- •CH_3_]^+•^. It was 28 Da lower than that of tetrahydropalmatine.

N-methylcanadine, compound 35, was detected as protonated molecular ions [M+2H]^+^. In MS^2^, this compound yielded ions at *m/z* 338 via a retro Diels–Alder (RDA) fragmentation reaction losing 16Da of oxygen (Figure 12 A, main text). The fragment ion at *m/z* 149 is possibly due to ring cleavage from the protoberberine skeleton (Deevanhxay et al., 2009). The MS^2^ fragment ion observed at *m/z* 190 in MS^2^ spectrum corresponds to the principal isoquinoline ion thus compound 88 was tentatively identified as edulitine. Fragment ions at *m/z* 334 [MH_2_O]^+^ and 318 [M-^•^ CH_3_- ^•^ H-H_2_O]^+^ were observed in MS^2^ supports a hydroxylation product of berberine thus compound 55 was identified as product ions i.e. dihydrosanguinarine. Compound 57 was thus identified as sanguinarine because it was two units (2 Da) less in its precursor ion. Compound 19 was identified as 6-hydroxy-10-methylsanguinarine with MS^1^ ion, [M+H]^+^ *m/z* 380. MS^2^ fragments 362[M+H-H_2_O]^+^ and *m/z* 275 [M+H-C_2_H_5_N -CO_2_]^+^ were formed. The compounds 19, 55 and 57 all have benzophenanthridine type skeleton of alkaloids. They have four benzene rings conjugated into the major skeleton, producing abundant fragments by neutral loss of substituents in the positive ion mode. Other compounds with benzophenanthridine type skeleton were compounds 6, 18, 20, 27, 40, 41, 42, 54, 56 and 63. Compounds 18, 20, and 42 were nitidines identified by comparing their MS/MS spectra with the published literature [21, 22] They were identified as 8-methoxy dihydronitidine (18, 20) and nitidine (compound 20). Compounds 6, 27, 40, 41, 54, 56 and 63 could be identified as chelerythrines by comparing their MS/MS spectra with earlier reports [22, 23]. Chelerythrine, compound 41 showed parent ion [M+H]^+^ at *m/z* 348. It eluted later than nitidine. The successive loss of CH_3_ (15 Da) and CO (28 Da) from the fragment ion *m/z* 317 could lead to the fragments *m/z* 290 [21]. Compound 27 was identified as oxychelerythrine. Compound 56, tentatively identified as, 8-O-demethylchelerythrine could give major fragment at *m/z* 319 due to the neutral loss of CH_3_ from M+H]^+^ ion at *m/z* 334 at N moiety [21]. Compound 54 had 2 Da more implying 2 hydrogen atoms and thus could be identified as 8-O-demethyldihydrochelerythrine and compound 63 is a product ion of 8-O-demethyldihyrochelerythrine identified as 9,10-didemethylchelerythrine [21]. Conjugation with a glucose could give 8-O-demethyl dihydrochelerythrine-9-glucoside, compound 6. Compound 39, showed 3 Da more than ion at *m/z* 348 of chelerythrine. The MS^2^ fragment ions at *m/z* 335 could be deduced by successive neutral loss of CH_3_ (15 Da) from N moeity from A ring of the ion at *m/z* 351. It was identified as fagaranine in line with literature [24]. Skimmianine (compound 82) showed [M+H]^+^ *m/z* 260 exhibited Q2 ion 245 and Q3 ion 202 after losing 15 Da in MS^2^ spectrum and a consecutive loss of 28 Da (CO) followed by a loss of 15 Da(CH_3_) with collision energy of 25, 40V respectively. Ribalinine, a dihydropyranoquinol-4-one, compound 83 however showed, Q2 ion 242 and Q3 ion 134 due to initial loss of a molecule of water followed by RDA cleavage through the N ring to give ion [M+ C_8_H_8_NO•]^+^. Its glucoside conjugate, compound 11, was detected after an extra 162 Da on ribalinine precursor ion and a methoxy derivative of this sugar conjuagate is compound 9 and 75 with extra 31 Da. Compounds 7 and 67 were tentatively identified as magnocurarine-6-glucoside and magnocurarine respectively. The MS^2^ fragments, *m/z* 314 and *m/z* 107 were common. The fragment ion at *m/z* 314 represents that of the aglycone magnocurarine while the ion at *m/z* 107 was observed in MS^2^ suggesting the existence of the benzene-ring mono-substitution by an OH group at C4^1^, in agreement with earlier literature[25, 26], thus they are benzylisoquinoline alkaloids. Another benzylisoquinoline alkaloid was compound 59, identified as reticuline. It was identified by their MS/MS spectral patterns of previous report [27]. Similarly, compound 29 is a benzylisoquinoline alkaloid and was identified as 8-methylisotembetarine when 14 Da was added to tembetarine (*m/z* 344) and the MS/MS spectrum similar to previous study [20]. Compound 90 was identified as 4-dihydro-6,7-dimethoxy- isoquinoline due to characteristic isoquinoline ion *m/z* 192 and major MS^2^ fragment *m/z* 177 ( [192-CH_3_]^+^) [20]. Similarly compound 29 had an isoquinoline skeleton and was identified as N-methyltetrahydrocolumbamine in comparison with previous report [27].

**References**

1. Fernández-Fernández R, López-Martínez JC, Romero-González R, Martínez-Vidal JL, Alarcón Flores MI, Garrido Frenich A. Simple LC–MS determination of citric and malic acids in fruits and vegetables. Chromatographia. 2010;72:55-62.

2. Ehling S, Cole S. Analysis of organic acids in fruit juices by liquid chromatography− mass spectrometry: an enhanced tool for authenticity testing. Journal of agricultural and food chemistry. 2011;59(6):2229-34.

3. Birkler RID, Støttrup NB, Hermannson S, Nielsen TT, Gregersen N, Bøtker HE, et al. A UPLC–MS/MS application for profiling of intermediary energy metabolites in microdialysis samples—A method for high-throughput. Journal of pharmaceutical and biomedical analysis. 2010;53(4):983-90.

4. Fang N, Yu S, Prior RL. LC/MS/MS characterization of phenolic constituents in dried plums. Journal of Agricultural and Food Chemistry. 2002;50(12):3579-85.

5. Owens JE, Holstege DM, Clifford AJ. High‐throughput adaptation for the quantitation of total folate in human red blood cells by LC‐MS/MS. Wiley Online Library; 2006.

6. Spínola V, Pinto J, Castilho PC. Identification and quantification of phenolic compounds of selected fruits from Madeira Island by HPLC-DAD–ESI-MSn and screening for their antioxidant activity. Food chemistry. 2015;173:14-30.

7. Rodríguez-Rivera MP, Lugo-Cervantes E, Winterhalter P, Jerz G. Metabolite profiling of polyphenols in peels of Citrus limetta Risso by combination of preparative high-speed countercurrent chromatography and LC–ESI–MS/MS. Food chemistry. 2014;158:139-52.

8. Rivera-Mondragón A, Tuenter E, Ortiz O, Sakavitsi ME, Nikou T, Halabalaki M, et al. UPLC-MS/MS-based molecular networking and NMR structural determination for the untargeted phytochemical characterization of the fruit of Crescentia cujete (Bignoniaceae). Phytochemistry. 2020;177:112438.

9. Park SY, Jung MY. UHPLC‐ESI‐MS/MS for the quantification of eight major Gingerols and Shogaols in ginger products: Effects of ionization polarity and mobile phase modifier on the sensitivity. Journal of Food Science. 2016;81(10):C2457-C65.

10. Jolad SD, Lantz RC, Solyom AM, Chen GJ, Bates RB, Timmermann BN. Fresh organically grown ginger (Zingiber officinale): composition and effects on LPS-induced PGE2 production. Phytochemistry. 2004;65(13):1937-54.

11. Asamenew G, Kim H-W, Lee M-K, Lee S-H, Kim YJ, Cha Y-S, et al. Characterization of phenolic compounds from normal ginger (Zingiber officinale Rosc.) and black ginger (Kaempferia parviflora Wall.) using UPLC–DAD–QToF–MS. European Food Research and Technology. 2019;245:653-65.

12. Sanz M, de Simón BF, Cadahía E, Esteruelas E, Muñoz AM, Hernández T, et al. LC‐DAD/ESI‐MS/MS study of phenolic compounds in ash (Fraxinus excelsior L. and F. americana L.) heartwood. Effect of toasting intensity at cooperage. Journal of Mass Spectrometry. 2012;47(7):905-18.

13. Tsimogiannis D, Samiotaki M, Panayotou G, Oreopoulou V. Characterization of flavonoid subgroups and hydroxy substitution by HPLC-MS/MS. Molecules. 2007;12(3):593-606.

14. Ngadjui BT, Tsopmo A, Ayafor JF, Connolly JD, Tamboue H. Hosloppin, a new pyrone-substituted flavonoid from Hoslundia opposita. Journal of Natural Products. 1995;58(1):109-11.

15. Ngadjui BT, Ayafor JF, Sondengam BL, Connolly JD, Rycroft DS, Tillequin F. Oppositin and 5-O-methylhoslundin, Pyrone-substituted Flavonoids of Hoslundia opposita. Phytochemistry. 1993;32(5):1313-5.

16. Taamalli A, Arráez‐Román D, Abaza L, Iswaldi I, Fernández‐Gutiérrez A, Zarrouk M, et al. LC‐MS‐based metabolite profiling of methanolic extracts from the medicinal and aromatic species Mentha pulegium and Origanum majorana. Phytochemical analysis. 2015;26(5):320-30.

17. Wang D, Wang J, Huang X, Tu Y, Ni K. Identification of polymethoxylated flavones from green tangerine peel (Pericarpium Citri Reticulatae Viride) by chromatographic and spectroscopic techniques. Journal of Pharmaceutical and Biomedical Analysis. 2007;44(1):63-9.

18. ReSpect MS/MS, RT Plant metabolomics database 2012 [updated 2012. Available from: <http://spectra.psc.riken.jp/>.

19. Yang W, Ye M, Liu M, Kong D, Shi R, Shi X, et al. A practical strategy for the characterization of coumarins in Radix Glehniae by liquid chromatography coupled with triple quadrupole-linear ion trap mass spectrometry. Journal of Chromatography A. 2010;1217(27):4587-600.

20. Le PM, McCooeye M, Windust A. Characterization of the alkaloids in goldenseal (Hydrastis canadensis) root by high resolution Orbitrap LC-MS n. Analytical and bioanalytical chemistry. 2013;405:4487-98.

21. Qing ZX, Cheng P, Liu XB, Liu YS, Zeng JG, Wang W. Structural speculation and identification of alkaloids in Macleaya cordata fruits by high‐performance liquid chromatography/quadrupole‐time‐of‐flight mass spectrometry combined with a screening procedure. Rapid Communications in Mass Spectrometry. 2014;28(9):1033-44.

22. Gathungu RM, Oldham JT, Bird SS, Lee-Parsons CW, Vouros P, Kautz R. Application of an integrated LC-UV-MS-NMR platform to the identification of secondary metabolites from cell cultures: benzophenanthridine alkaloids from elicited Eschscholzia californica (california poppy) cell cultures. Analytical Methods. 2012;4(5):1315-25.

23. Liang M, Zhang W, Hu J, Liu R, Zhang C. Simultaneous analysis of alkaloids from Zanthoxylum nitidum by high performance liquid chromatography–diode array detector–electrospray tandem mass spectrometry. Journal of Pharmaceutical and Biomedical Analysis. 2006;42(2):178-83.

24. Jia C-P, Huang X-L, Li Y, Feng F. Analysis of alkaloids in Zanthoxylum nitidum by HPLC-DAD/ESI-Q-TOF-MS. Zhongguo Zhong yao za zhi= Zhongguo Zhongyao Zazhi= China Journal of Chinese Materia Medica. 2013;38(8):1198-202.

25. Oger J-M, Fardeau A, Richomme P, Guinaudeau H, Fournet A. Nouveaux alcaloïdes isoquinoléiques isolés d'une Lauraceae bolivienne: Aniba canelilla HBK. Canadian journal of chemistry. 1993;71(8):1128-35.

26. Lee S-S, Lin Y-J, Chen C-K, Liu KC, Chen C-H. Quaternary alkaloids from Litsea cubeba and Cryptocarya konishii. Journal of Natural Products. 1993;56(11):1971-6.

27. Deevanhxay P, Suzuki M, Maeshibu N, Li H, Tanaka K, Hirose S. Simultaneous characterization of quaternary alkaloids, 8-oxoprotoberberine alkaloids, and a steroid compound in Coscinium fenestratum by liquid chromatography hybrid ion trap time-of-flight mass spectrometry. Journal of pharmaceutical and biomedical analysis. 2009;50(3):413-25.
